# Facilitators and barriers to COVID-19 vaccination among healthcare workers and the general population in Guinea

**DOI:** 10.1186/s12879-022-07742-3

**Published:** 2022-09-27

**Authors:** Almamy Amara Toure, Fodé Amara Traore, Gnoume Camara, Aboubacar Sidiki Magassouba, Ibrahima Barry, Mohamed Lamine Kourouma, Younoussa Sylla, Naby Yaya Conte, Diao Cisse, Nafissatou Dioubaté, Sidikiba Sidibe, Abdoul Habib Beavogui, Alexandre Delamou

**Affiliations:** 1 National Centre of Training and Recherche in Rural Health of Mafèrinyah, BP 2649, Forécariah, Guinea; 2 Department of Public Health, Kofi Annan University of Guinea, Conakry, Guinea; 3 Faculté des Sciences et Techniques de la Santé, Centre Hospitalo-Universitaire de Conakry, Service de Maladies Infectieuses, Université Gamal Abdel Nasser, Conakry, Guinea; 4 Agence Nationale de Sécurité Sanitaire, Conakry, Guinea; 5 Department of Public Health, Faculty of Sciences and Health Techniques, Gamal Abdel Nasser University, Conakry, Guinea; 6 Centre MURAZ, Bobo-Dioulasso, Burkina Faso; 7 African Centre of Excellence in the Prevention and Control of Communicable Diseases (CEA-PCMT), Conakry, Guinea

**Keywords:** COVID-19 vaccination, Healthcare workers, General population, Barriers, Facilitators, Guinea

## Abstract

**Introduction:**

The advent of the effective COVID-19 vaccine was the most eagerly expected worldwide. However, this hope quickly became hesitation and denial in many countries, including Guinea. Understanding the reasons for low vaccine coverage is essential to achieving herd immunity leading to disease control. This study aimed to comprehend the facilitators and barriers to the acceptance COVID-19 vaccine in Guinea.

**Methods:**

The survey focused on healthcare workers (HCWs) and the general population (GP) in 4 natural regions in Guinea from 23 March 2021 to 25 August 2021. We used the Fishbein integration model to study the behaviours of HWCs and GP regarding vaccination. A mixed cross-sectional study collected knowledge, attitudes, norms, and perceptions. Regression and thematic content analysis identified the main facilitators and barriers to vaccination.

**Results:**

We surveyed 3547 HCWs and 3663 GP. The proportion of people vaccinated was 65% among HCWs and 31% among the GP. For HCWs: the main factors associated with vaccination against COVID-19 were as follows: absence of pregnancy AOR = 4.65 [3.23–6.78], being supportive of vaccination AOR = 1.94 [1.66–2.27] and being an adult AOR = 1.64 [1.26–2.16]. Regarding the GP, the following factors increased the odds of vaccination: absence of pregnancy AOR = 1.93 [CI 1.01–3.91], being favourable for vaccination AOR = 3.48 [CI 2.91–4.17], being an adult AOR = 1.72 [CI 1.38–2.14] and being able to get the vaccine AOR = 4.67 [CI 3.76–5.84]. Semi-interviews revealed fear, lack of trust, and hesitant perception of the government as potential barriers to vaccination.

**Conclusion:**

This study suggests that beliefs and negative perceptions are potential barriers to vaccination against COVID-19 among HCWs and the GP. Policies should emphasise practical strategies to mitigate these barriers among young people and pregnant women. Lastly, there is a need to improve access to vaccines in the GP.

**Supplementary Information:**

The online version contains supplementary material available at 10.1186/s12879-022-07742-3.

## Introduction

In March 2020, Guinea reported its first case of COVID-19. As a result, on 13 April 2022, 6540 cases, with 441 deaths and 36,054 recoveries, were recorded [1]. In the wake of COVID-19, Government undertook many actions to limit the spread of the epidemic. One crucial step was to comfort the population that restrictive measures were required. The Guinean population, firmly devoted to religions, could never believe that disease could lead to the closure of places of worship (mosques and churches). The fear induced by that environment, especially the world was unarmed as to a specific therapy, is the bottom line that fuelled anxiety and stress among the population.

Therefore, the hope hinged on making effective vaccines to stop the spread of the disease. Vaccine development in trials usually takes a long time, from 2 to 15 years [2, 3]. However, global mobilisation supported that expectation to make the dream possible [4] Thus, that context shortened this process to less than 24 months [2, 3]. This speed in manufacturing would be likely to instil doubt in the beliefs of the population regarding vaccination.

Moreover, misinformation through the media has taken hold in many parts of the world, including Africa [5] Yet, a few months earlier, a study had predicted a favourable opinion of vaccination in sub-Saharan Africa [6] Indeed more than 70% of the participants in this survey were willing to be vaccinated when the vaccine became available, and 60% had confidence in developing the vaccine [6].

As misinformation, fear of backlash and uncertainty spread to many countries, the concept of hesitancy has increasingly found its way into the literature [7, 8] highlighting the delay in being vaccinated against COVID-19 vaccination. Those attitudes varied from one region to another, some being favourable (high rate to be vaccinated) and others unfavourable (low vaccination rates) [9]. Against this shared opinion, Guinea initiated its first vaccination in March 2021. Healthcare workers (HCWs) were the first to be vaccinated. The latter target deserves special attention in the battle against epidemics; some studies have shown significant hesitation among HCWs to get vaccinated. [10–12]

Achieving herd immunity is imperative in vaccination to reduce transmission and disease impact [13, 14]. Studies show that a minimum of 67% is required to achieve this goal [13–15]. However, Guinea struggles to reach 30% of the people vaccinated. Since vaccination is free, it is essential to understand the reasons for this low commitment. Previous studies have focused on a particular target to explain the causes of non-vaccination [7, 10, 11, 16–18].

Additionally, almost all those studies have looked at the intention to vaccinate. Yet, changes in opinion can occur from one period to another, as Wang et al. showed in a survey of differences in willingness to vaccinate [19]. Given the interaction between the general population (GP) and HCWs and the possibility that the HCWs could mobilise the GP. Understanding the reasons for low vaccine coverage is essential to achieving herd immunity leading to disease control. The primary objective of this study was to comprehend the facilitators and barriers to COVID-19 vaccine acceptance in Guinea.

## Methodology

### Study setting

We conducted the study in the four natural regions of Guinea (Lower Guinea, Middle Guinea, Upper Guinea and Forest Guinea). Latitude North 7° 30′ and 12° 30′ Longitude West: 8° and 15°, with an area of 245,857 km^2^ and 12,907,395 [20]. The bordered countries are Guinea-Bissau, Senegal, Mali, Ivory Coast, Sierra Leone, and Liberia [20] The National Health Security Agency leads the vaccination program against COVID-19 in Guinea with the following vaccines Sinovac, AstraZeneca, Pfizer, Sputnik1; Sputnik2; Johnson and Johnson. At the start of vaccination (more than 6 months) in Guinea, only two vaccines were available, including Sputnik and Sinovac.

### Study type and period

We conducted a mixed cross-sectional study. The quantitative survey focused on knowledge, attitudes and perception about COVID-19, and the qualitative to explain the quantitative findings. The study occurred from 23 March 2021 to 25 August 2021.

### Study population

For the diversity of opinion, we targeted the general population (GP) and health care workers (HCWs).

### Selection criteria

#### Inclusion criteria

- Free and informed consent;
- At least 18 years old at the time of inclusion;
- Available and able to express themselves.

#### Exclusion criteria

- Refusal to participate in the survey;

### Sampling

- Selection method
  The selection was made at two different levels.
  **Health care workers** Among the 300 operational health facilities provided by the ministry of health, we randomly selected 150 health facilities within districts of the regions. We then used proportional allocation to choose the required HCWs and gradually included them in each facility.
  **The general population** We had 400 workplaces (public, private and schools) provided by the districts’ authorities. We randomly selected 200 of them and then used proportional allocation to choose the number of people required and gradually included them in each workplace.
- Sample size
  Quantitative component
    **Health care workers** We hypothesised that 70% of health care workers favour vaccination across Africa [6]. With the desired precision of 5%, the sample size was calculated using the formula. **N** = $\frac{{\varvec{Z}^{\mathbf{2}}}({\varvec{P}}\boldsymbol{*}{\varvec{Q}})}{{\varvec{i}}^{\mathbf{2}}}$ [21]. The minimum expected size is 322; considering the 10% non-response rates, this size was increased to 370 per health district in the region.
    **General population.** We hypothesised that 50% of the population favour vaccination [6] The formula calculates the sample size with the desired precision of 5%. **N** = $\frac{{\varvec{Z}^{\mathbf{2}}}({\varvec{P}}\boldsymbol{*}{\varvec{Q}})}{{\varvec{i}^{\mathbf{2}}}}$ [21]. The expected minimum size is 384; considering the 10% non-response rates, this size will increase to 420 per prefecture in the natural region.
  Qualitative component
    Participants from the HCWs and the general population were interviewed in each area of the natural region selected. We interviewed 20 participants among the HCWs for the four natural sites and 50 participants among the general population, i.e. 70 participants per region. As a result, we included 280 participants. The participants for the qualitative survey were from the respondents to the quantitative part; if necessary, other participants were selected from the same sites.

### Survey implementation

1. Training of the data collectors
  The interviewers (independent data collectors) were recruited and trained in the survey’s methodology, the collection tools and the collection technique.
2. Pilot study
  We conducted a pilot study to pre-test the tools focused on ONA platform utilisation, form ‘coherence through android, and checking and sending the finalised filled forms. That step allowed us to assess the feasibility of the field survey. We targeted the unselected areas with similar characteristics to those of the selected regions. In the pilot areas, we chose 50 people, including 25 from HCWs and 25 from the GP.
3. Data collection
  Investigators used Android phones to administer the questionnaires to participants at the workplace or by appointment at the nearest or most convenient location. The data was recorded through an Android application (ODK) downloaded and connected to the ONA server (https://ona.io/home/). ODK is used to integrate the online format of the study questionnaire, and ONA platform monitored the data collecting process through the dynamic dashboard. We used a semi-structured interview for the qualitative part, and the participants’ permission was obtained to record the interview.

### Theoretical framework and variables

**Fig. 1:**
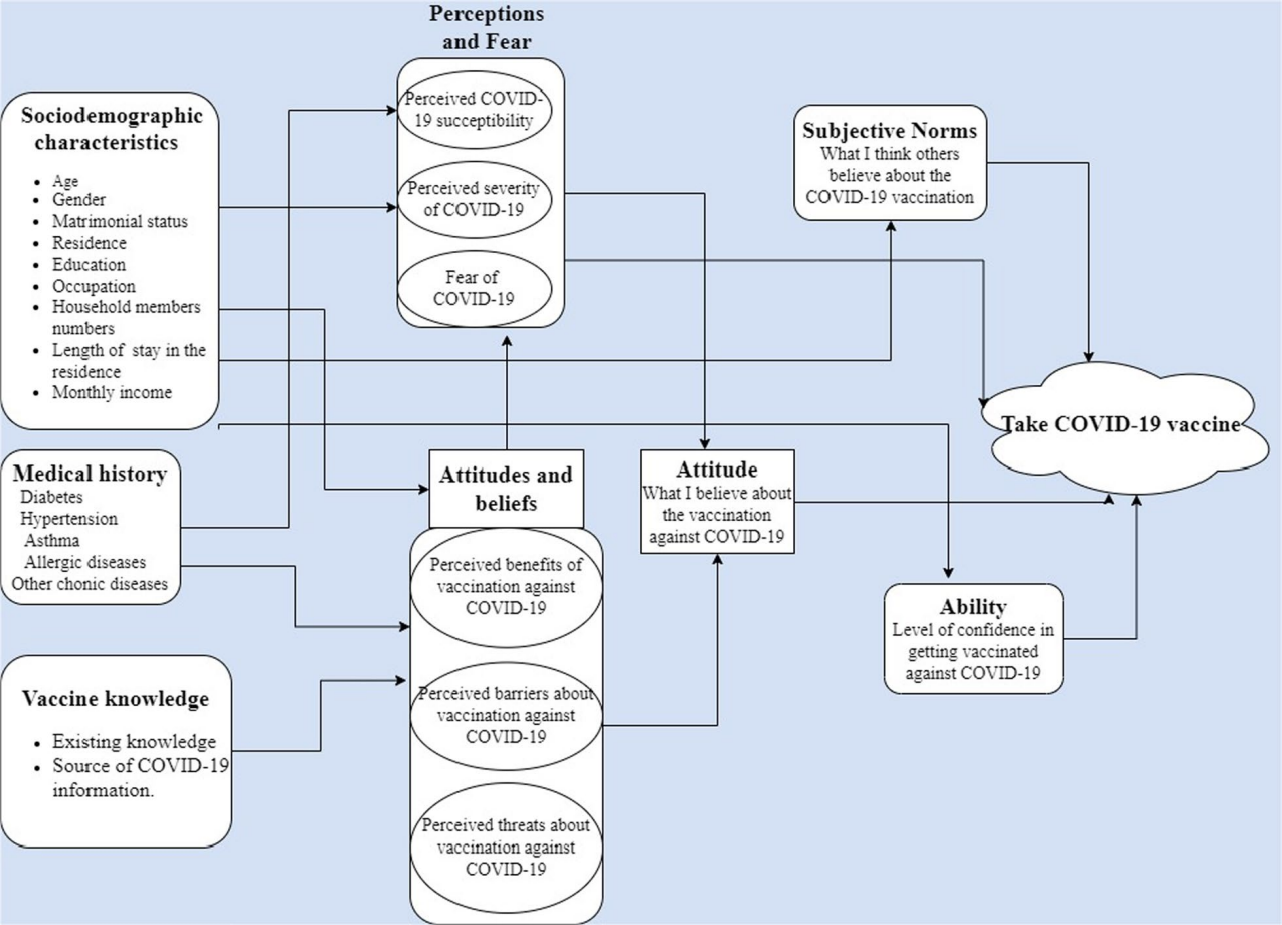
Theoretical framework of the study

The theoretical framework is based on the Fishbein integration model [22]. The elicitation or preliminary analysis relies on a literature review. Our model was adapted from existing work [23–25]. The model presented in Fig. 1 incorporates socio-demographic characteristics, disease history, vaccination information, disease perception and apprehension, and barriers. These different elements influence the individual’s attitude. Finally, norms and the ability to be vaccinated potentiate the previous factors to predict vaccination against COVID-19.

### Study variables

A. **Dependent variable** The participant’s vaccination status. The participants were asked about their vaccination status. Whether or not the participant is vaccinated.
B. **Independent variables**
  **Socio-demographic items** Age: in completed years; sex represented by male and female, marital status (single, married); residence; the level of education; occupation (job held by the participant), the number of persons in the household, number of persons with age ≥ to 18 years, the duration of stay in the place of residence (less than 6 months, more than 6 months), monthly income and current pregnancy.
  **Medical conditions** Diseases reported by the participant: asthma, hypertension, diabetes, obesity or overweight, allergic diseases (sinusitis, rhinitis, severe adverse reactions to drugs), and other chronic diseases. COVID-19 and vaccination items are summarised in Additional file 1: Table S1.
  *Quality control and assurance* We put a system in place strict monitoring of the fieldwork progress, including the geolocation of interviewers. A data manager checked the internal consistency and validity of the data daily. Any inconsistencies were reported and dealt with them. Finally, we have set parameters in the form that help to prevent the occurrence of missing data by data collectors.
  *Statistical analysis* For the following variables: perception/fear, attitudes/beliefs, subjective norms, ability and intention to receive the vaccine, we classified them according to the average of the scale scores [26] Thus, participants with scores above or equal to the mean were considered positive perceptions. Otherwise, the perception was negative. For attitude and belief, we divided into two parts: items related to negative attitude (when the score is lower than the mean, the attitude is less negative; otherwise, the attitude is more negative) and those related to positive attitude (when the score is lower than average, the attitude is less positive; otherwise, the attitude is more positive). For norms, when the score of the scales was below average, the norms are considered favourable; otherwise, the norms are unfavourable). When the score was below average for ability, the participants are deemed unable; otherwise, they are able.) Finally, when the score was below the mean of income for the intention, the participants had less intention to be vaccinated; otherwise, they had more intention to be vaccinated. Quantitative variables were analysed using the median and interquartile range, and qualitative variables using the percentage. We considered households with high income when the mean income is ≥ 2000000GNF and the number of people in the household is ≤ 10. For low-income households, when the mean income is < 2000000GNF and the number of people in the household is > 10, all other cases were considered middle income. The Chi-square or Fisher test and the Student or Wilcoxon test were used for the descriptive analysis. We used multivariate logistic regression between the participants’ vaccination status and the independent variables to identify facilitators and barriers. Then, we put in the classification and regression tree (CART) the significant variables of the previous regression models for the HCWs and the general population while keeping the dependent variable. The last analysis was backed by qualitative research with the thematic content method. The statistical tests were considered significant at the risk α = 0.005. We used the software R version 4.1.2 and Stata 15 to analyse all the data.

## Results

### A) Quantitative component

**Fig. 2:**
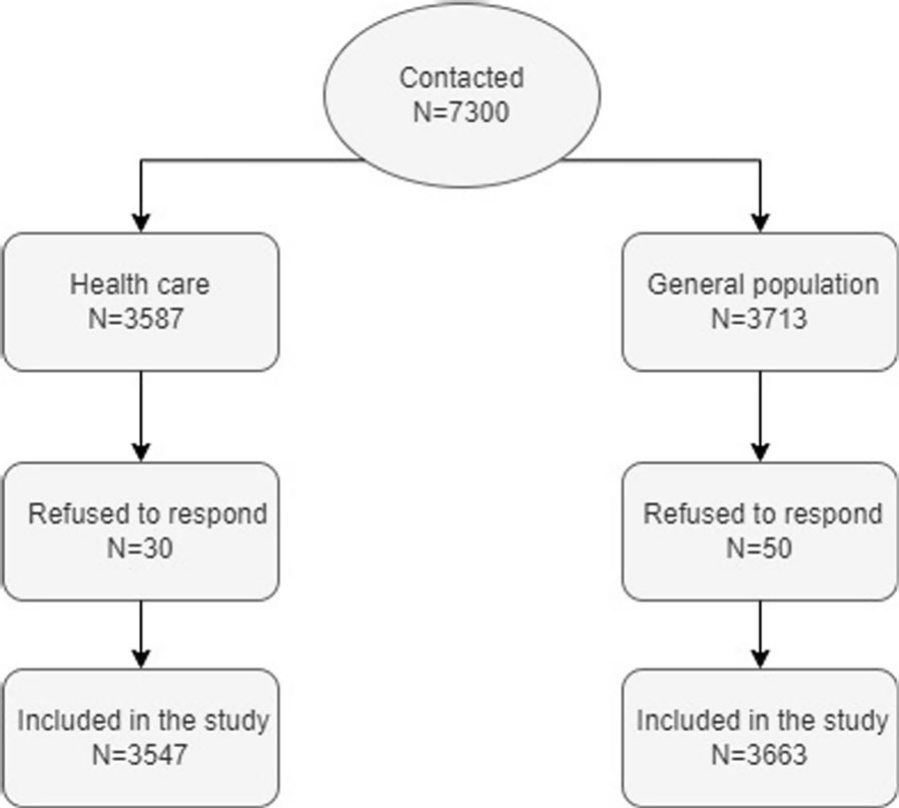
Inclusion flow diagram

Figure 2 shows the flow of inclusion among HCWs and the GP with 7210 participants.

#### Descriptive analysis

Additional file 1: Tables S2–S4 show the description of the study sample. We surveyed 3547 HCWs and 3663 of the general population. The general knowledge of HCWs about vaccination was 45%, while that of the general population was 48%. For HCWs and the general population, the overall perception of good intention was 49% and 48%, respectively. The positive attitude was 73% for HCWs and only 16% for the general population.

**Fig. 3:**
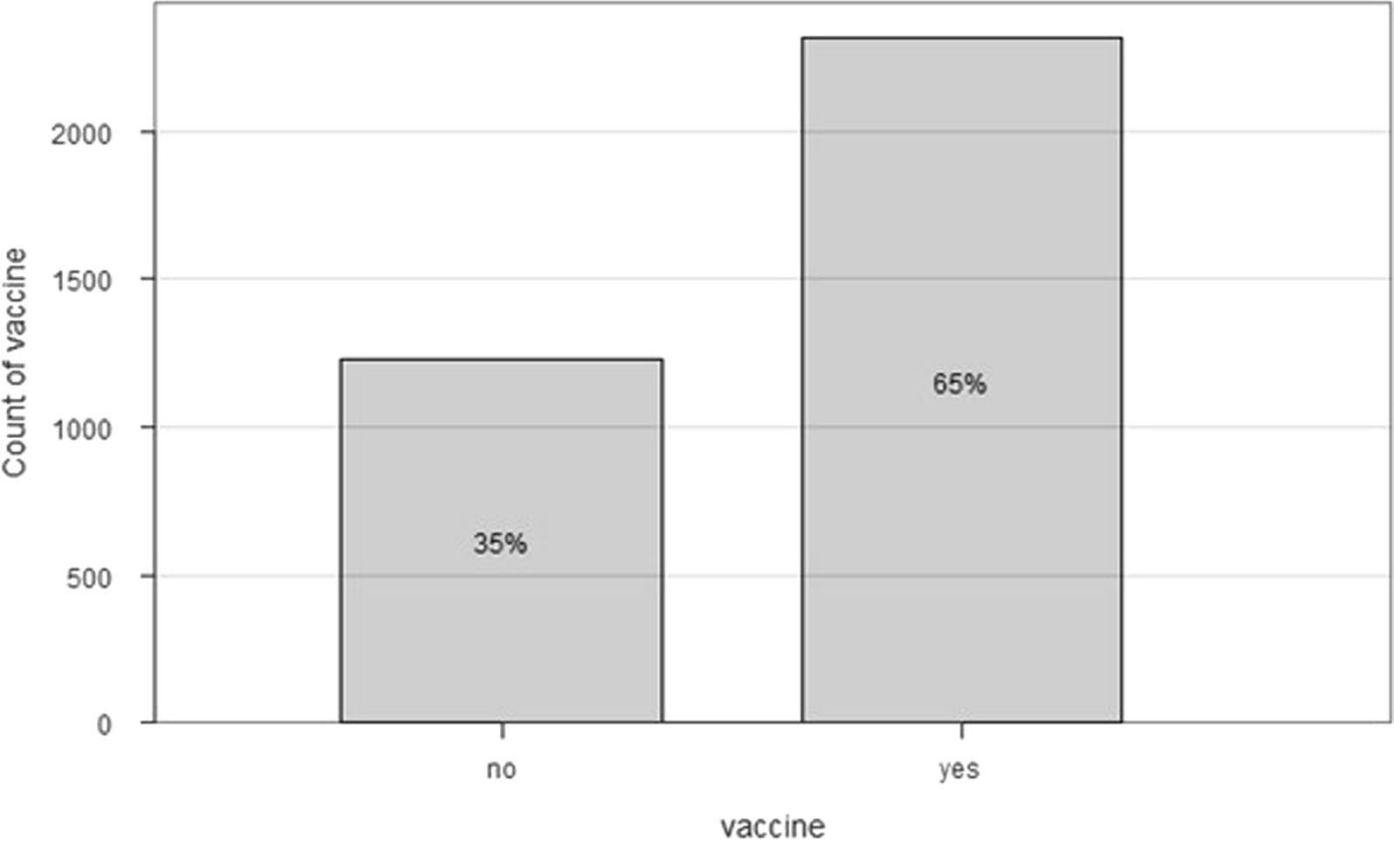
The healthcare workers who get vaccinated from March to August 2021. N = 3547

**Fig. 4:**
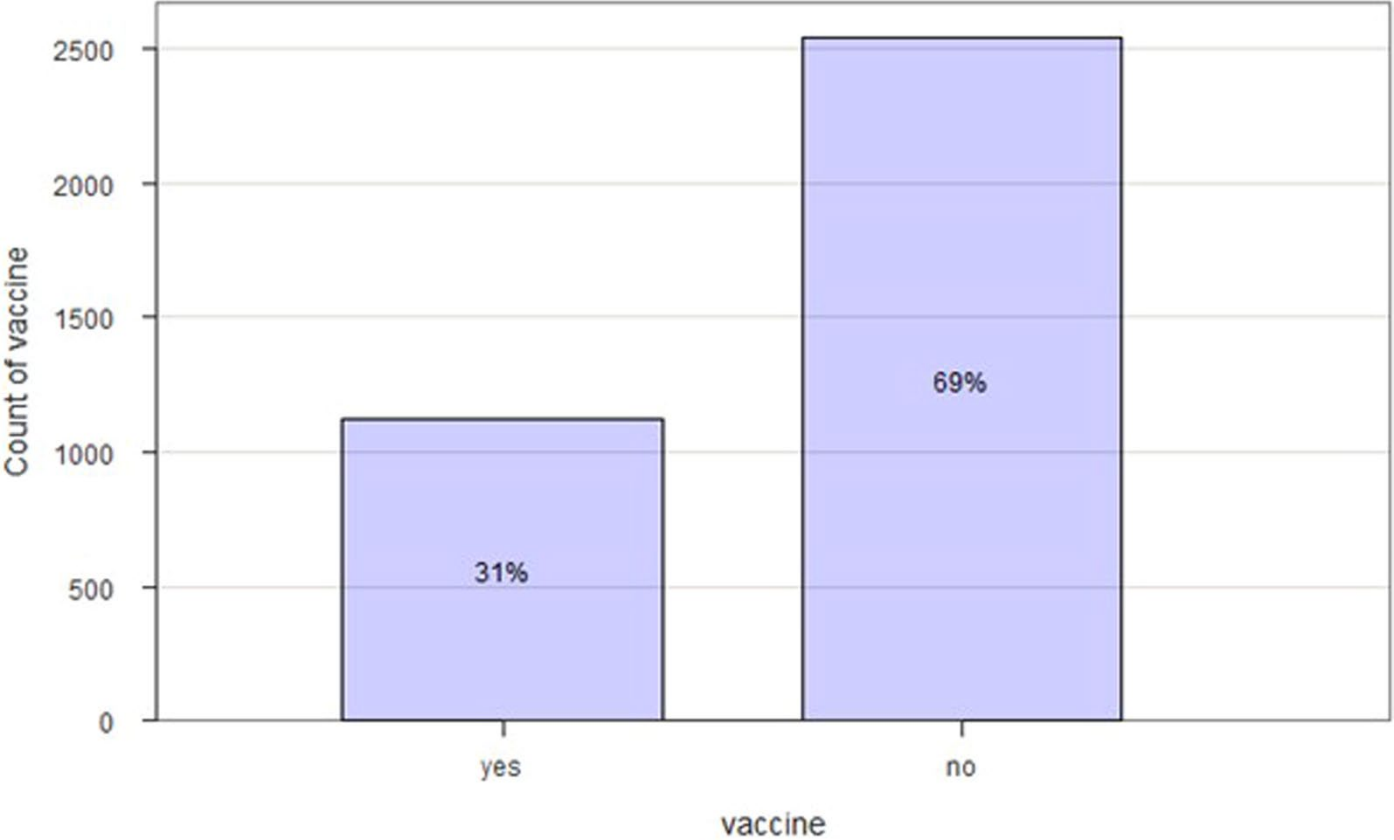
The general population who get vaccinated from March to August 2021. N = 3663

**Fig. 5:**
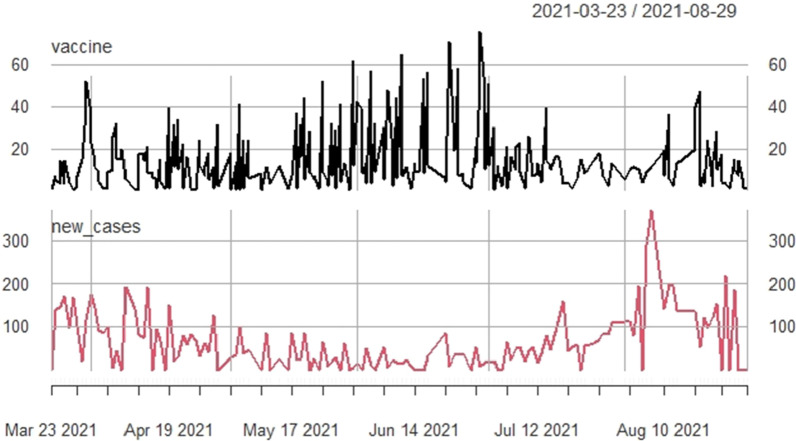
Evolution of vaccination against COVID-19 and COVID-19 cases

Furthermore, the proportion of people vaccinated was 65% among HCWs and 31% among the general population (Figs. 3 and 4). Figure 5 shows the evolution of vaccination with COVID-19 cases. We noticed that from April to August, the number of people vaccinated increased, and the cases of COVID-19 decreased.

**Fig. 6:**
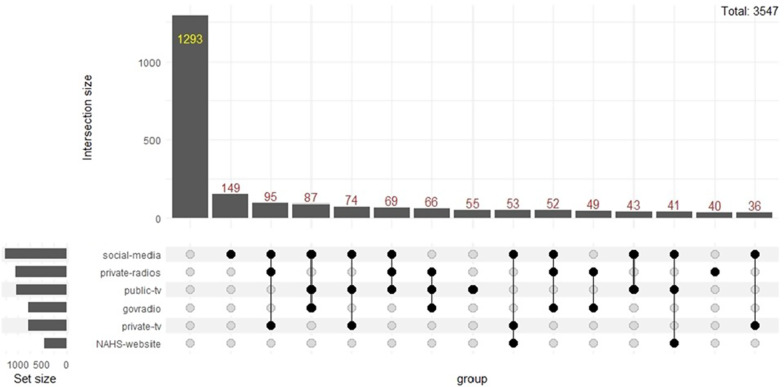
COVID-19 Source of News for the healthcare workers

**Fig. 7:**
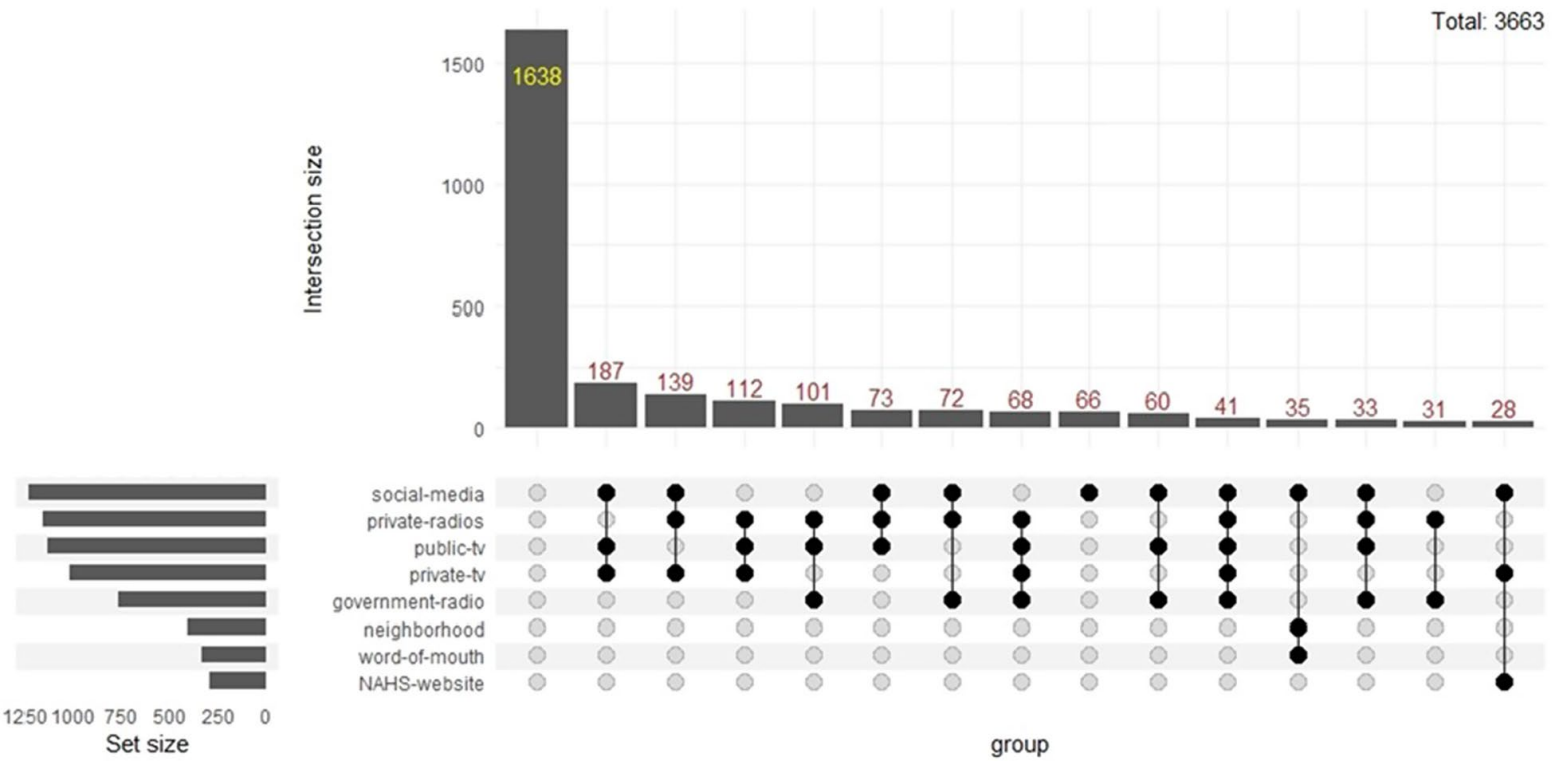
COVID-19 Source of News for the general population

HCWs’ three sources of information concerning COVID19 were social networks, private radio and national television (Fig. 6). The three most used sources simultaneously were social networks, private radio and private television (Fig. 6). The three primary sources of information used for the general population were social networks, private radio, and national television (Fig. 7). The primary sources of information used simultaneously were social media, public television, and private television (Fig. 7).

#### Univariate analysis

Table 1 shows the factors associated with vaccination among HCWs, which were as follows: gender, marital status, education level, occupation, pregnancy, being hypertensive, knowledge of the vaccine, seeking information about COVID-19 in the last 3 days of the survey, positive perception of the COVID-19 vaccine, positive and negative attitude, norms, household income, intention to vaccinate and age. We found the same observations in the general population besides perception and the factors listed, such as the length of stay in the residential setting, history of diabetes and obesity, and ability to have the COVID-19 vaccine.

**Table 1:** Univariate analysis: factors associated with COVID-19 vaccination

	Health care workers			General population		
	Already get vaccinated			Already get vaccinated		
	No N = 1231	Yes N = 2316	p-value^a^	No N = 2542	Yes N = 1121	p-value^a^
Socio-demographic factors						
Age			< 0.001**			< 0.001**
Young	1145 (93%)	1970 (85%)		2148 (85%)	754 (67%)	
Adult	86 (7.0%)	346 (15%)		394 (15%)	367 (33%)	
Gender			< 0.001**			0.008*
Men	407 (33%)	932 (40%)		1730 (68%)	812 (72%)	
Women	824 (67%)	1384 (60%)		812 (32%)	309 (28%)	
Matrimonial status			< 0.001**			< 0.001**
Married	615 (50%)	1320 (57%)		1065 (42%)	646 (58%)	
Single	616 (50%)	996 (43%)		1477 (58%)	475 (42%)	
Éducation			< 0.001**			0.003*
Secondary	52 (4.2%)	50 (2.2%)		1243 (49%)	481 (43%)	
University	333 (27%)	781 (34%)		1155 (45%)	561 (50%)	
High school	846 (69%)	1485 (64%)		144 (5.7%)	79 (7.0%)	
Occupation			< 0.001**			< 0.001**
Nurse assistant	706 (57%)	1170 (51%)				
Laboratory technician	51 (4.1%)	114 (4.9%)				
Physician	222 (18%)	572 (25%)				
Medical support	49 (4.0%)	46 (2.0%)				
Midwife	153 (12%)	317 (14%)				
Internship	50 (4.1%)	97 (4.2%)				
Private-employee				156 (6.1%)	104 (9.3%)	
Student				723 (28%)	173 (15%)	
Civil-servant				446 (18%)	387 (35%)	
Freelance				1053 (41%)	381 (34%)	
Unemployed				164 (6.5%)	76 (6.8%)	
Household size			0.4^ns^			0.7^ns^
[1, 5]	561 (46%)	1001 (43%)		1227 (48%)	526 (47%)	
[5, 10]	483 (39%)	945 (41%)		934 (37%)	428 (38%)	
[10, 30]	187 (15%)	370 (16%)		381 (15%)	167 (15%)	
≥ to 18 years old	3 (2, 5)	3 (2, 5)	0.11^ns^	3 (2, 5)	3 (2, 5)	0.6^ns^
Length-stay			0.057^ns^			< 0.001**
< 6 months	142 (12%)	220 (9.5%)		314 (12%)	90 (8.0%)	
≥ 6 months	1089 (88%)	2096 (91%)		2228 (88%)	1031 (92%)	
Pregnancy			< 0.001**			0.006*
Yes	102 (8.3%)	50 (2.2%)		63 (2.5%)	14 (1.2%)	
No	721 (59%)	1333 (58%)		746 (29%)	294 (26%)	
Not applicable	408 (33%)	933 (40%)		1733 (68%)	813 (73%)	
Household income			< 0.001**			0.020*
High income	53 (4.3%)	213 (9.2%)		282 (11%)	160 (14%)	
Low income	172 (14%)	340 (15%)		339 (13%)	136 (12%)	
Middle income	1006 (82%)	1763 (76%)		1921 (76%)	825 (74%)	
Medical conditions						
Diabetes						
Yes	25 (2.0%)	58 (2.5%)	0.4^ns^	59 (2.3%)	55 (4.9%)	< 0.001**
No	1206 (98%)	2258 (97.5)		2483 (97.7)	1066 (95.1)	
Hypertension						
Yes	38 (3.1%)	116 (5.0%)	0.008*	120 (4.7%)	129 (12%)	< 0.001**
No	1193 (96.9%)	2200 (95%)		2422 (96.3%)	992 (88%)	
Obesity						
Yes	202 (16%)	411 (18%)	0.3^ns^	582 (23%)	315 (28%)	< 0.001**
No	1029 (84%)	820 (82%)		1960 (77%)	806 (82%)	
Asthma						
Yes	43 (3.5%)	80 (3.5%)	> 0.9^ns^	72 (2.8%)	31 (2.8%)	> 0.9^ns^
No	1180 (96.5%)	2236 (96.5%)		2470 (97.2%)	1090 (97.2%)	
Other allergic conditions						
Yes	231 (19%)	433 (19%)	> 0.9^ns^	449 (18%)	200 (18%)	0.9^ns^
No	1000 (81%)	1883 (81%)		2093 (82%)	921 (82%)	
Other chronic diseases						
Yes	105 (8.5%)	231 (10.0%)	0.2^ns^	239 (9.4%)	103 (9.2%)	0.8
No	1126 (91.5%)	2085 (90%)		2303 (81.6%)	1018 (81.8%)	
COVID-19 factors related						
Vaccine knowledge						
Yes	692 (56%)	917 (40%)	< 0.001**	1083 (43%)	686 (61%)	< 0.001**
No	539 (44%)	1399 (60%)		1459 (57%)	435 (39%)	
Seeking COVID vaccine news in the last 3 days						
Yes	734 (60%)	1520 (66%)	< 0.001**	1288 (51%)	737 (66%)	< 0.001**
No	497 (40%)	796 (44%)		1254 (49%)	384 (44%)	
Perception			0.002*			0.4^ns^
Positive	562 (46%)	1184 (51%)		1243 (49%)	531 (47%)	
Negative	669 (54%)	1132 (49%)		1299 (51%)	590 (53%)	
Negative			< 0.001**			< 0.001**
Less negative	563 (46%)	1328 (57%)		1286 (51%)	675 (60%)	
More negative	668 (54%)	988 (43%)		1256 (49%)	446 (40%)	
Positive attitude			0.024*			< 0.001**
Less positive	875 (71%)	1728 (75%)		498 (20%)	99 (8.8%)	
More positive	356 (29%)	588 (25%)		2044 (80%)	1022 (91%)	
Norm			< 0.001**			< 0.001**
Less favourable	676 (55%)	1016 (44%)		1616 (64%)	311 (28%)	
More favourable	555 (45%)	1300 (56%)		926 (36%)	810 (72%)	
Ability to get the vaccine			0.9^ns^			< 0.001**
Not able	1071 (87%)	2010 (87%)		1213 (48%)	127 (11%)	
Able	160 (13%)	306 (13%)		1329 (52%)	994 (89%)	
Intend to get vaccinated			< 0.001**			0.2^ ns^
Less intend	732 (59%)	1669 (72%)		1398 (55%)	641 (57%)	
More intend	499 (41%)	647 (28%)		1144 (45%)	480 (43%)	

^a^Wilcoxon rank sum test; Pearson’s Chi-squared test

^ns^Non significant; *significant; **very significant

#### Multivariate analysis

Table 2 shows the factors associated with vaccination against COVID-19 in multivariate analysis. For HCWs. Single people were 30% less likely to get vaccinated than married people, AOR = 0.70 [CI 0.60–0.82]; those with high school levels were 75% more likely to get vaccinated than those with secondary school, AOR = 1.75 [CI 1.13–2.70]; medical support worker were 52% less likely to get vaccinated compared to nurses, AOR = 0.48 [CI 0.29–0.78], while midwives were 32% likelier to get vaccinated compared to nurses, AOR = 1.32 [CI 1.04:1.67]. Non-pregnant women had 4.65 odds of being vaccinated than pregnant women, AOR = 4.65 [CI 3.23–6.78]. Those with higher vaccine knowledge were 38% less likely to get vaccinated than those with lower vaccine knowledge, AOR = 0.62 [CI 0.53–0.72]. Those with a more negative attitude were 36% less likely to get vaccinated, AO = 0.64 [CI 0.55–0.75], than those with less. Those favourable to vaccination were 94% more likely to get vaccinated than those less, AOR = 1.94 [CI 1.66–2.27]. Participants who had not looked for information on COVID-19 for 3 days were 16% less likely to get vaccinated than those who did it, AOR = 0.84 [CI 0.71–0.98]. Participants with more intention to get vaccinated were 50% less likely than those with less it, AOR = 0.50 [CI 0.42–0.59]. Those with a middle household income were 38% less likely to get vaccinated than those with high income, AOR = 0.62 [CI 0.44–0.86]. Finally, adults were 64% more likely to get vaccinated than young people, AOR = 1.64 [CI 1.26–2.16].

**Table 2:** Multivariate analysis: factors associated with vaccination against COVID-19

Characteristic	Health care workers			General population		
	OR	95% CI	p-value	OR	95% CI	p-value
Age						
Young	–	–		–	–	
Adult	1.64	1.26, 2.16	< 0.001**	1.72	1.38, 2.14	< 0.001**
Matrimonial status						
Married	–	–				
Single	0.70	0.60, 0.82	< 0.001**			
Education						
Secondary	–	–		–	–	
University	1.56	0.95, 2.57	0.078^ns^	1.48	1.22, 1.80	< 0.001**
High school	1.75	1.13, 2.70	0.012*	1.33	0.95, 1.87	0.10
Occupation						
Nurse assistant	–	–				
Laboratory technician	1.05	0.68, 1.64	0.8^ns^			
Physician	0.99	0.69, 1.42	> 0.9^ns^			
Medical support	0.48	0.29, 0.78	0.003*			
Midwife	1.32	1.04, 1.67	0.022*			
Internship	1.00	0.69, 1.47	> 0.9^ns^			
Private-employee				–	–	
Student				0.47	0.33, 0.68	< 0.001**
Civil-servant				1.32	0.95, 1.83	0.10
Freelance				0.81	0.59, 1.12	0.2
Unemployed				0.78	0.51, 1.18	0.2
Pregnancy						
Yes	–	–		–	–	
No	4.65	3.23, 6.78	< 0.001**	1.93	1.01, 3.91	0.055*
Vaccine knowledge						
No	–	–		0.66	0.55, 0.78	< 0.001*
Yes	0.62	0.53, 0.72	< 0.001**	–	–	
Seeking COVID vaccine news in last 3 days						
Yes	–	–				
No	0.84	0.71, 0.98	0.027*			
Negative attitude						
Less negative	–	–		–	–	
Much negative	0.64	0.55, 0.75	< 0.001*	0.73	0.61, 0.86	< 0.001**
Positive attitude						
Less positive	–	–		–	–	
Much positive	0.84	0.71, 1.00	0.050^ns^	1.77	1.36, 2.31	< 0.001**
Norms						
Less favourable	–	–		–	–	
Favourable	1.94	1.66, 2.27	< 0.001**	3.48	2.91, 4.17	< 0.001**
Intend to get vaccinated						
Less intend	–	–		–	–	
More intend	0.50	0.42, 0.59	< 0.001**	0.44	0.37, 0.52	< 0.001**
Household income						
High income	–	–				
Low income	0.74	0.51, 1.08	0.12^ns^			
Middle income	0.62	0.44, 0.86	0.006*			
≥ to 18 years old				0.98	0.95, 1.01	0.13^ns^
Length-stay						
< 6 months				–	–	
≥ 6 months				1.31	0.99, 1.74	0.063^ns^
Hypertension						
Yes				–	–	
No				0.59	0.43, 0.82	0.002**
Obesity						
Yes				–	–	
No				0.81	0.67, 0.98	0.032*
Other chronic disease						
Yes				–	–	
No				1.36	1.03, 1.81	0.034*
Perception						
Positive				–	–	
Negative				0.81	0.68, 0.96	0.014*
Ability to get vaccine						
Not able				–	–	
Able				4.67	3.76, 5.84	< 0.001**

*OR* odds ratio, *CI* confidence interval

^ns^Non significant; *significant; **very significant

As for the general population, non-pregnant women were 93% more likely to get vaccinated than pregnant women, AOR = 1.93 [CI 1.01–3.91]. Those with insufficient knowledge about vaccines were 34% less likely to vaccinate than those with more knowledge, AOR = 0.66 [CI 0.55–0.78]. Those with more negative attitudes were 27% less likely to vaccinate than those with less negative attitudes AOR = 0.73 [CI 0.61–0.86]. Participants with more positive attitudes were 16% less likely to vaccinate than those with less positive attitudes AOR = 1.77 [CI 1.36–2.31]. Those favourable to vaccination were 3.48 times more likely to get vaccinated than those less it, AOR = 3.48 [CI 2.91–4.17]. Participants with more intention to get vaccinated were 56% less likely than those with less it, AOR = 0.44 [CI 0.37–0.52]. Adults were 72% more likely vaccinated than young people, AOR = 1.72 [CI 1.38–2.14]. Those without hypertension were 41% less likely to get vaccinated than those with hypertension, AOR = 0.59 [CI 0.43–0.82]. Similarly, those who were not obese were 19% less likely to get vaccinated than those who were obese. Participants with other chronic diseases were 36% more likely to get vaccinated than those without; AOR = 1.36 [CI 1.03–1.81]. Those with a negative perception were 19% less likely to get vaccinated than those with a positive AOR = 0.81 [CI 0.68, 0.96]. Finally, those who could get the vaccine were 4.67 times more likely to get vaccinated than those unable AOR = 4.67 [CI 3.76–5.84].

#### Classification and regression tree (CART)

**Fig. 8:**
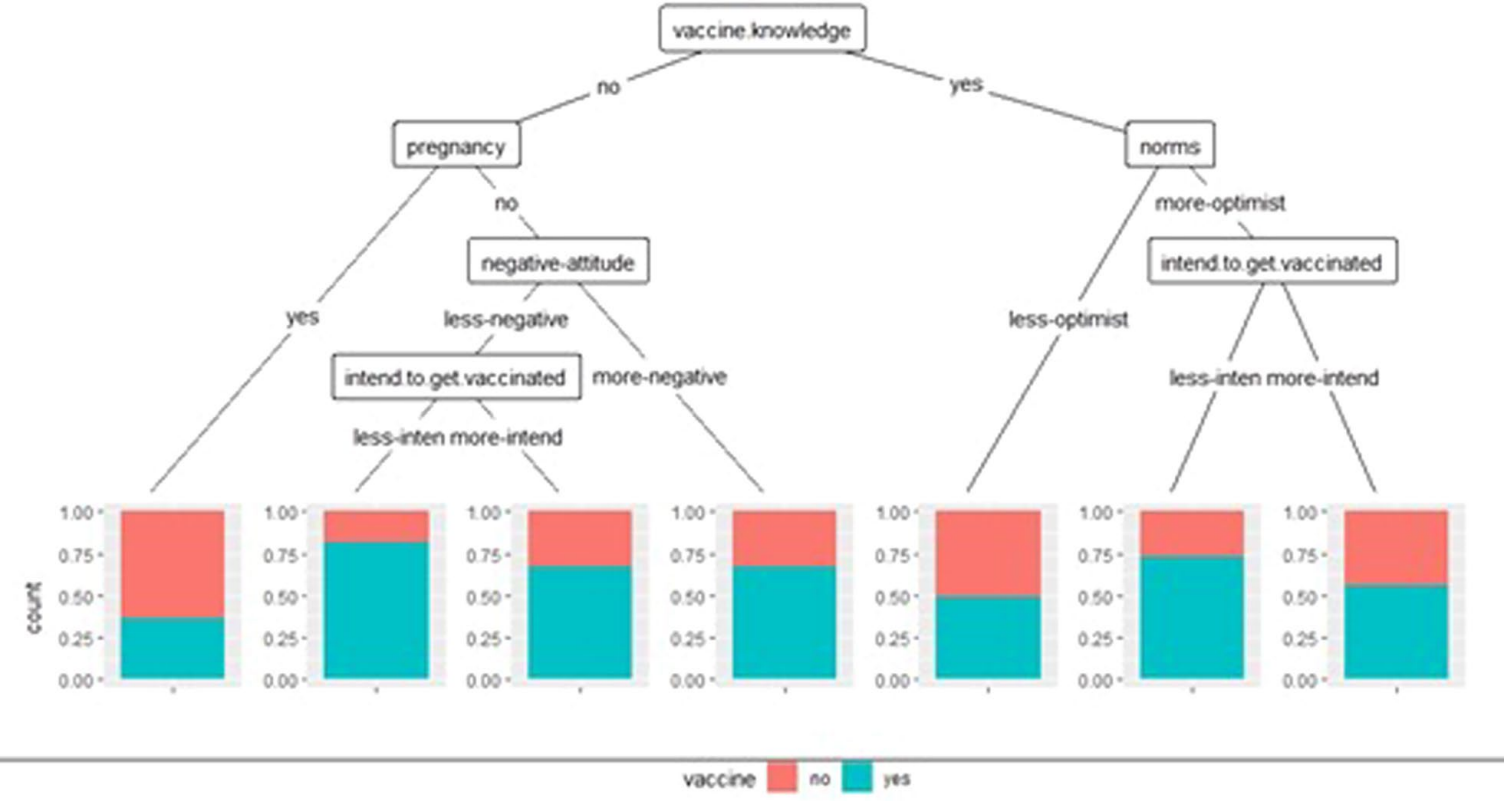
CART. Factors associated with vaccination against COVID-19 among healthcare workers

**Fig. 9:**
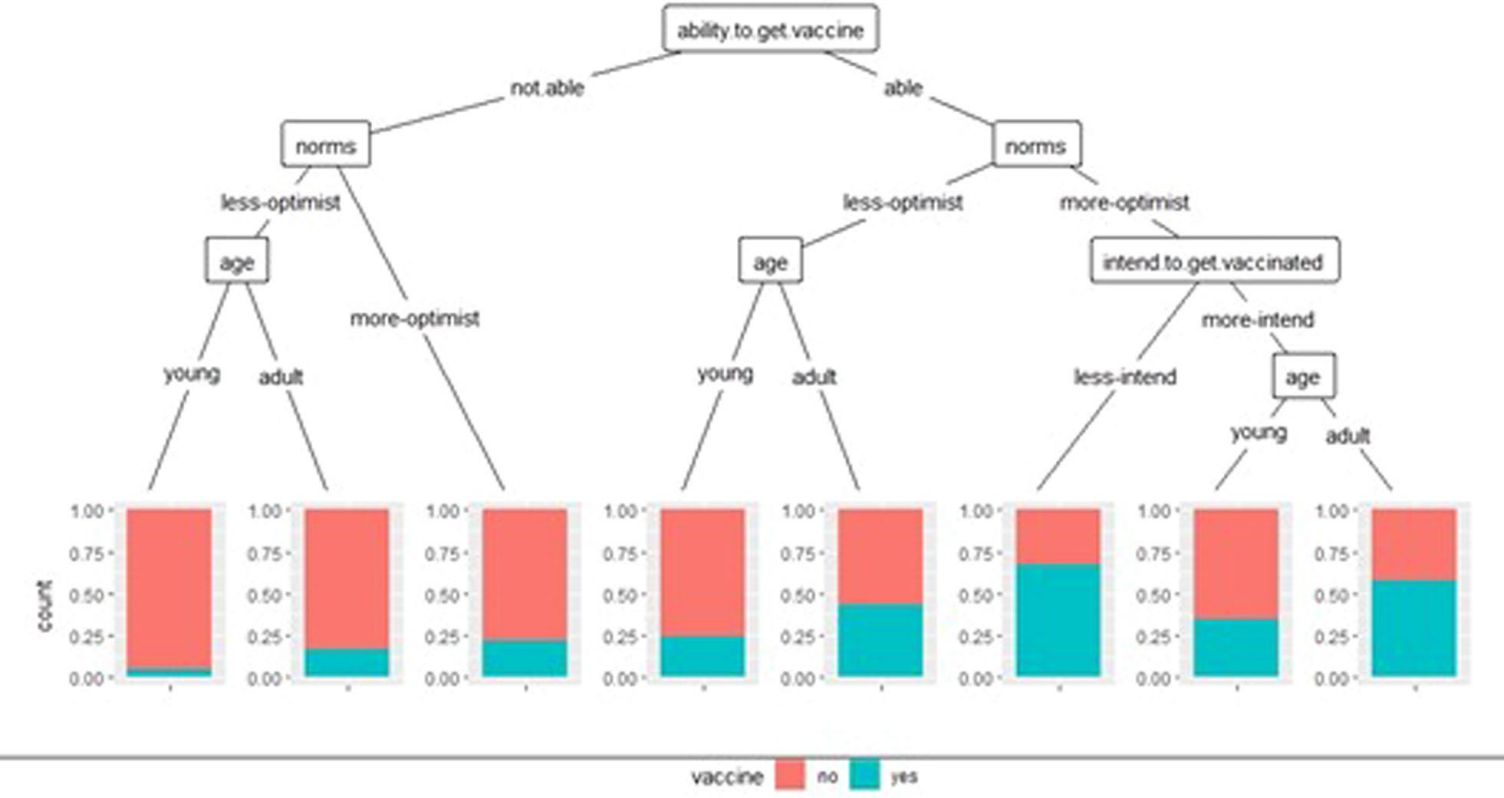
CART. Factors associated with vaccination against COVID-19 in the general population. NB: young: whose age is < 40 and adult: whose age is ≥ 40

The regression tree in Figs. 8 and 9 for the HCWs and the general population shows the association of the dependent variable with the significant variables in the multivariate logistic regression models. Overall, the general knowledge of the vaccine discriminates against COVID-19 vaccination among HCWs. According to this variable, two categories can be specified then into seven classes:

Four classes stand out in the first category (low knowledge about vaccines): pregnant and non-pregnant women. It can be seen that whatever the attitude, the proportion of people vaccinated is higher for non-pregnant women than for pregnant women.

The norms are divided into three groups in the 2nd category (good knowledge of vaccination). We found that the most favourable participants for vaccination had higher proportions than those who were less.

**General population** The ability to obtain the vaccine is the main factor determining vaccination against COVID19. Three main segments stand out. Those with favourable norms for vaccination were more likely to get vaccinated against COVID-19. The middle segment tells us that adults were more likely to vaccinate if they were less. In the third segment, where the models favour vaccination, young people were less likely to get vaccinated than others.

### B) Qualitative component

#### Theme 1. knowledge of COVID-19 and the vaccine

##### Subtheme 1_1. Knowledge of the signs of the disease and the types of vaccines against COVID-19

This study shows that the COVID-19 disease and the vaccines used today against this disease seem to be unknown in the GP. The simple reason that justifies this observation is that most of them have not been able to satisfy the main signs of covid 19 and the types of vaccine available in Guinea. For the following questions, what do you know about covid 19? What are available vaccines used in the fight against covid 19 in Guinea? Some interviewees in the General population (GP) let us hear the following: *“Covid 19 is a deadly disease that manifests itself through hematemesis and coughing”* [Man 29 years old, GP]. Another adds: “*coughing, fever and malaria are the symptoms of* COVID-19” [36-year-old man, GP].

On the other hand, the caregivers had a good knowledge of the disease. One expressed himself: “COVID-19 *is a very contagious and deadly disease due to the coronavirus. It mainly attacks the respiratory system and can go from a simple cold to severe pneumonia. It is transmitted by air, contact with soiled objects, and human-to-human. It is a disease that began in 2019 in China and spread worldwide, creating a pandemic*” [31-year-old man, Doctor]. In the same logic within the GP, testimonies collected from our interviewees lead us to believe their limit in the knowledge of vaccines. One of them expresses himself: “*I don’t know the name of the germ responsible for covid, but for the vaccine, there are many such as vaccines for the Chinese, there recently but I don’t know their scientific names. There is also a German vaccine, a Russian vaccine…*” [31-year-old man, GP].

##### Subtheme 1_2. Knowledge of the methods of transmission and means of prevention of COVID-19

Some factors such as the severity of the disease, the modes of communication and the means of prevention were major elements that emerged from most interviewees’ comments. To justify this passage, here is the testimony of an interviewee: “*It is a severe disease, it is a fatal disease, now to prevent this disease we must respect the barrier measures, avoid shaking hands, wear masks, avoid going anywhere where there are a lot of people. The mode of transmission is to avoid shaking hands, used disinfectants every time*” [Woman 42 years old, GP].

##### Subtheme1_3. Knowledge of information sources

For almost all of our respondents, the primary sources of information mentioned were traditional sources from the health authorities and their partners (radio, television and awareness campaigns in public places). Through the question, have you ever heard of vaccination against COVID-19? If So, through what sources have you heard about it? The answers were almost uniform “*yes, of course, it’s the news; we hear about it all day long through the media, TV, radio, magazines, articles, word of mouth through many sources*” [Woman 31 years old, GP]. Another added: “*Yes, we make the massage on television and the radios. Some people attend the compounds for awareness-raising*” [36-year-old man, GP]. However, other sources of information, such as the internet and the written press, were more widely known by health care workers and those with a certain level of education within the GP.

#### Theme 2. The attitude of the population to vaccines and vaccination

Opinions remain divergent at this point: Within the GP, some would have a belief that affects their decision not to go to be vaccinated. The simple fact questioned the vaccine’s effectiveness: it was possible to contract the disease even after being vaccinated. “*This vaccine is ineffective; otherwise, how can we be vaccinated and when we do the test again, we still tell you that you have covid, I don’t believe it” [Man 30 years old, GP]. Another defends her refusal in these terms “I refused to be vaccinated against Covid-19 because I don’t have complete confidence in the vaccine. Because of the major side effects that can happen in the short and long term, I don’t know what it can cause in the body*” [Woman 31 years old, GP].

We also noticed this lack of confidence in the vaccine. An interviewee lets us hear the following*: *“*No, I took it, but I don’t trust it, I took it as a doctor, but I don’t trust it*” [25-year-old man, Doctor].

Another important aspect is the influence of certain family members or their entourage, through their words and titles, negatively influence the thinking or attitude of the GP members. According to some interviewees, they might disagree with their family’s decision but would be forced to accept to maintain family ties. Others come to explain their experiences after taking the vaccine, thus creating a psychosis within the GP “*… For example, there is another when they take the vaccine, and they get sick. These are the same people who will tell others, don’t go to get vaccinated. When I went there the other time, that’s what I got. So, it can influence your decision to go for the vaccination. So, if you are not someone who has faith and confidence, you are not going to go…*” [28-year-old man, GP].

#### Theme 3. Perception of the population about the existence of covid

From the point of view of their belief in the disease, perceptions remain very divided. Some interviewees are convinced that this disease is a matter planned by the government. According to them, it is a method to control the population, especially in this period of political instability. Thus, when asked about this, one of the interviewees expressed himself in these terms*: *“*Mr, I reassure you that this disease is not valid; it is a matter of politics. You know, it’s a way to prevent protests. As we know, when we talk about the disease, it’s an argument for not having a grouping. So you understand what is specific in Guinea is politics; by proof, the authorities vaccinate in their office, we don’t even know what they are taking there.*” [Man 38 years old, GP].

On the other hand, although healthcare workers recognise some limitations of the vaccine, the vast majority believe that the availability of a vaccine would be beneficial for the population and hope that rapid ramp-up will reduce the frequency of the disease. He describes the GP’s abstention from vaccination as ignorance on their behalf: “*I think that this disease is true, and besides, it is not only in Guinea that it is facing …. Of course some do not believe, but I think it is ignorance that does this. And I hope that taking the vaccines will lead to a decrease in the disease. Otherwise, a disease that affects almost every country in the world deserves to be believed…*” [30-year-old man, Doctor].

#### Theme 4. Factors facilitating vaccination among the population

Thus, called to give their point of view on the aspect facilitating the acceptance of vaccination by the population, our respondents believe that the main factor remains effective awareness and good communication. According to these participants, awareness-raising and good communication through radio, television, or other media sources with the involvement of government authorities would reduce the GP’s distrust of the vaccine. Given the importance of social media, communication would be essential to promote vaccination as a measure to prevent contagion. Thus, one participant lets us hear the following: *“This is effective awareness-raising; nothing can go without it; there must be real communication among the population. A country with 80% of illiterates must resort to awareness-raising; it is necessary to communicate much about the disease and the vaccine, which the Guineans have missed a lot. And in addition, it must be done in all languages and every day*” [Man 30 years old, GP].

Some interviewees believe that the involvement of credible people (religious and well renowned) can promote adherence to vaccination. “*religious leaders, people who are popular in the GP when they are involved in raising awareness in communication and setting an example to the population”. They can get people to join because they have the population’s trust*” [39-year-old man, GP].

Following the same logic, others believe it would be necessary for government and health authorities and their families to use the public places that the average population frequents for the same purposes. According to them, the absence of rulers at the level of vaccination centres means discrimination against the GP. The type of vaccine and the manufacturer’s country affect the acceptability of the vaccine. In these words, an interviewee lets us hear: “*Tried to break this discrimination. That the senior managers of this country come to be vaccinated in the same health centres as the GP, that everyone sees them and that they take the same type of vaccine as the rest of the population will create a climate of trust. That’s enough to say that we see a minister coming here to be vaccinated; it’s not us ordinary citizens who will doubt that now. It is said that even the minister came to take his vaccine in this health centre. Therefore, just like that, it stimulates the population and gives more credibility to the vaccinator. This is a first-class act of bravery to encourage the population not to doubt. That’s the trigger in a way*” [29-year-old woman, GP]. A second added, *“Well, I’m not afraid to take the vaccine, but I have to see those in front of the authorities and the government get vaccinated as well as all their families so that others can join”* [26-year-old man, GP].

#### Theme 5. Factors limiting vaccination among the population

Some elements restricting the vaccination to the breasts of the interviewees emerged during our interview.

Despite having some knowledge about most aspects of the disease and the vaccine, most healthcare workers stressed the non-compliance with the health authorities’ commitment to the vaccination program. According to them, the non-compliance with the vaccination schedule following a stock out in the first doses would have discouraged some candidates for vaccination, as this neurology doctor testifies to us.

“*I would say that perhaps the non-compliance with the vaccination schedule due to a shortage of vaccine stock was the basis for this discouragement because when I took my first vaccine, we were told that the second dose would be in two weeks. But it’s beyond that; we had to wait because the vaccine was unavailable. So that’s what demoralised a lot of people. In addition to the non-compliance with this program, the waiting time for the second dose was long. It lasted a month so that we could reschedule for the second dose. Even if it is currently available, in the beginning, there was a difficulty.*” [Woman 32 years old, Nurse]

A second crucial aspect was the lack of confidence that the population had in their government and the vaccine. According to them, the authority has made a difference in vaccine distribution, leading to distrust and a lack of trust. This lack of confidence lies in the type of vaccines; the Russian for the administration and the Chinese for the GP. “*The only reason I’m going to say this is that people don’t trust the government. By the way, politics plays a role, that’s the main reason you tell people to get vaccinated, and they refuse to do it. In addition, they’re still there saying that the vaccine the government took is not the same as for the population, you see, huh! “[Man 30 years old, GP]. Another adds: “the only peculiarity is that in Guinea here we had two types of vaccine, the one produced by the Russians and the one made by China, and at the time, there were rumours that the population benefited from one subtype of vaccine and the government from another, that is to say, that the Russian vaccine was given to government staff and we beat it, it was the Chinese vaccine so just for that already we have ideas in our heads; is the vaccine we received the suitable vaccine? Why gave the population one type of vaccine, and they only benefited from another type of vaccine when we are all in the same country? That’s kind of it; there is a controversy at this level, and I doubt even if I had to get vaccinated, that’s it.*” [Man 40 years old, GP].

A third important aspect was the fear of side effects and health risks. Within the GP, some participants reported an inadequate communication approach during the covid 19 awareness campaign. According to them, this campaign focused on two fundamental aspects: the disease’s severity and the benefits of vaccination. A critical element was ignored during the campaign, such as the side effects related to the vaccine.

*“The first thing was the origin of the vaccine as the government benefited from one type of vaccine (Russian) and another type (Chinese), so it was already causing fear in my level because we know that Chinese products are not of good quality. I was afraid of reacting to the vaccine or developing other pathologies because I am in a neurology department where we have seen reactions from people who developed secondary myelitis to vaccines. They take the vaccine, and soon after, there is a paralysis that sets in it is rare, but it is something that cannot be ruled out. Similarly, sources of information claim that some people have developed other pathologies secondary to vaccination …”* [25-year-old man, Doctor]. Further, another makes us live his perception “*…, as they have already shown on TV. We see it in social networks when you take the Astra Zenica vaccine, it can cause allergic reactions. The other time they said that it has side effects that can lead to blood clotting. People can be afraid that the same thing will manifest itself on my person, it’s because of that otherwise …”* [GP 35 years old].

## Discussion

The COVID-19 pandemic has left hundreds of families in Guinea in mourning. Hence, people expected the advent of an effective vaccine. The burden of this disease declined populations worldwide into unprecedented fear and worry. Preventive measures such as vaccination should be seen as beneficial. However, we found that the proportion of people vaccinated is lower than anticipated.

More than half of the HCWs were vaccinated, with only a small proportion of the general population. Yet, a herd immunity of over 67% would favour a reduction in infection [13, 14]. Our findings are similar to some studies about the intention to get vaccinated [10, 16, 27]. This low proportion of people vaccinated contrasts with the reality that indicates that vaccination works in our context. Indeed, by looking at the evolution of the vaccination status with the number of COVID-19 cases during the study period, we can see the effect of the vaccination (Fig. 5). We found that single people were less likely to be vaccinated than married people for HCWs. In a society that emphasises wedding and family responsibility, single people tend to be young and less likely to take responsibility. These observations corroborate our study’s finding that adults were more likely to get vaccinated. Most HCWs in our setting are high school students who often care for patients and are at greater risk of exposure. The medical support staff are administrators, pharmacists, and other support staff who are not involved in the care. They may therefore be prone to delay their vaccination. Pregnant HCWs were less likely to be vaccinated than non-pregnant healthcare workers. The initial policy to countries clearly stated that pregnant women should not be vaccinated, regardless of their level of exposure. However, the vaccines used then, such as SINOVAC (inactivated), were recommended during pregnancy. Moreover, recent studies indicate the tolerance of the vaccine in pregnant women with mRNA [28, 29].

We also identify that increased knowledge of vaccines or vaccination resulted in a high probability of HCWs not being vaccinated. This outcome contrasts but is not surprising as the qualitative analysis reveals that some HCWs are vaccinated only by conviction. Trust in authority is essential for promoting adherence to vaccination [18, 27].

Alternatively, a favourable opinion increases the probability of vaccination. We noted in this study that HCWs’ knowledge was insufficient to predict an increase in the proportion of people vaccinated. The study shows that those who had not looked for updated information for more than 3 days were less likely to get vaccinated. We noted that those who had a good intention towards vaccination were less likely to get vaccinated; the reason is trivial; as seen in the previous study [18], it is possible to have goodwill initially and later on not to get vaccinated for various reasons.

As for the GP, the factors associated with vaccination against COVID-19 were diverse. As with the HCWs, non-pregnant women were more likely to be vaccinated than pregnant women, and the same reason for the HCWs remains valid here. We found that, similar to healthcare workers, and negative beliefs reduced the likelihood of being vaccinated. According to them, the absence of the authorities at the vaccination centres means discrimination against the community. Hence, the choice of the vaccine type and manufacturer country are the precursors of the vaccine’s acceptability.

Contrary to previous realities, favourable opinion increases the probability of vaccination in the population. Other essential elements are identified at the level of the GP, i.e. the history of illnesses that increase the likelihood of vaccination. This attitude would be part of a preventive action among these people to avoid complications of their health condition with COVID-19. Finally, the population without the vaccine or easy access to it was less likely to be vaccinated. We noticed vaccination sites were initially limited and stood for great challenges for those who had to move from one place to another and pay the transportation fees.

Our study is one of the first to combine healthcare workers and the general population. It also incorporates a mixed methodology that explains Guinea’s barriers and facilitators to COVID-19 vaccination. Our study is representative of the natural regions of Guinea, whose socio-cultural characteristics remain diverse. This inference is limited to active people in the workplace, not household members. We considered that working people accounted for an active part of the population and were most at risk as they interacted with others. It would have been even more interesting in this study if we had studied the factors associated with a vaccination before and during vaccination. The two targets are not comparable but offer the possibility of extracting hypotheses about the variability of specific characteristics that may be of standard or opposite interest—for example, the ability to have a significant portion of the population vaccinated through HCWs. We dichotomized the scales of the COVID-19 and vaccination variables; this leads to a loss of information but produces a more straightforward picture to classify behaviours. Finally, in any cross-sectional study, we cannot prove causality.

## Conclusion

The simultaneous study of healthcare workers and the general population shows different perspectives regarding vaccination against COVID-19. The main facilitators of COVID-19 vaccine acceptance among HCWs were as follows: high education level, being not pregnant, good vaccine knowledge, being favourable to vaccination, and increased income, while the barriers were: being single, being a medical support worker, having a negative attitude, youth and having more intention to get vaccinated. Regarding the GP, the following factors were the facilitators of the COVID-19 vaccine acceptance: being not pregnant, being favourable to vaccination, having hypertension, having a positive attitude, having chronic diseases, and being able to get a vaccine, while the barriers were: a negative attitude, youth. Vaccine access must be expedited for HCWs and GP, especially pregnant women and youth. Finally, we must identify effective communication strategies to reduce negative attitudes.

## Supplementary Information

**Additional file 1.** Supplementary material.

**Additional file 2.** Dataset supporting the conclusions of this article.

**Additional file 3.** Dataset supporting the conclusions of this article.

**Additional file 4.** Dataset supporting the conclusions of this article.

**Additional file 5.** Dataset supporting the conclusions of this article.

**Additional file 6.** Dataset supporting the conclusions of this article.

## Data Availability

The dataset supporting the conclusions of this article is included within the article and its Additional files 2, 3, 4, 5, and 6

## Abbreviations

AOR: Adjusted odds ratio
CART: Classification and Regression Trees
GP: General population
HCWs: Healthcare workers
WHO: World Health Organization
CI: Confidence interval
CNFRSR: Centre National de Formation et de Recherce en Santé Rurale de Mafèrinyah
