# Supplementary material for: Facilitators and barriers to COVID-19 vaccination among healthcare workers and the general population in Guinea
Source: BMC Infect Dis. 2022 Sep 27;22:752. doi: 10.1186/s12879-022-07742-3 (PMC9514191; doi:10.1186/s12879-022-07742-3)
Supplement: Supplementary file 1 — Additional file 1. Supplementary material. [file 12879_2022_7742_MOESM1_ESM.docx]

**Facilitators and barriers to COVID-19 vaccination among healthcare workers and the general population in Guinea.**

Almamy Amara TOURE^1,2^, Fodé Amara TRAORE^3,4^, Gnoume CAMARA3, Aboubacar Sidiki MAGASSOUBA^5^, Ibrahima BARRY^1^, Mohamed Lamine KOUROUMA^4^, Younoussa SYLLA^1^, Naby Yaya CONTE^1,5^, Diao CISSE5, Nafissatou DIOUBATÉ^1^, Sidikiba SIDIBE^6,7^, Abdoul Habib BEAVOGUI1, Alexandre DELAMOU^5,6^.

**Authors' affiliations**
1 Maferinyah National Centre for Training and Research in Rural Health (CNFRSR), Forécariah, Guinea. 2 Kofi Annan University of Guinea.Conakry,Guinea. 3 Centre Hospitalo-Universitaire de Conakry,Service de maladies infectieuses. 4 Agence Nationale de Sécurité Sanitaire,Conakry,Guinée. 5 Centre MURAZ, Burkina Faso.6 Department of Public Health, Faculty of Sciences and Health Techniques, Gamal Abdel Nasser University, Conakry, Guinea. 7 African Centre of Excellence in the Prevention and Control of communicable Diseases (CEA-PCMT).

**Additional tables**

**Table S1. COVID-19 and vaccination items**

| **Items** | **Questions used** | **Scale/response** |
| --- | --- | --- |
| **knowledge about vaccination** | **-Healthcare workers:**  vaccine definition  vaccine types  post-injection adverse effects  individual and herd immunity  **-General population:**  general principle of vaccination | Yes/no  Yes/no  Yes/no  Yes/no  Yes/no |
| **Information sources** | Have you recently sought news about COVID-19? | **Yes/no** |
| **Perception/fear of COVID-19:**   - **Perceived Susceptibility of the disease** | I am likely to get COVID-19  I am at risk of COVID-19  I may get COVID-19 | **1** strongly disagree  2 disagree  **3** neutral  **4** agree  5 strongly agree |
| **Perceived seriousness of the disease** | **I think that COVID-19 is a severe health problem**  I believe that COVID-19 has negative consequences  I think that COVID-19 is highly harmful | 1 strongly disagree  2 disagree  3 neutral  4 agree  5 strongly agree |
| **Fear of COVID** | I am terrified of COVID-19  Thinking about COVID-19 makes me feel uncomfortable  My palms get sweaty when I think about COVID-19  Watching the news about COVID-19 on social networks makes me nervous or anxious  I cannot sleep because I am fearful of catching COVID-19  My heart rate increases, or I have palpitations when I think about COVID-19 | 1 strongly disagree  2 disagree  3 neutral  4 agree  5 strongly agree |
| **Attitudes and beliefs:**  **-Positive attitude**  **-Negative attitude**  **-Negative attitude** | Taking COVID-19 vaccination will help prevent coronavirus  Taking COVID-19 vaccine will help strengthen the body’s immunity in fighting viruses  A vaccine is reserved for a group of people  A vaccine is too expensive for me  A procedure to get the vaccine is laborious  I am afraid of the side effects of the vaccine  I heard that the vaccine induces the disease  Vaccine is made to prevent reproduction  I don’t trust vaccines | 1 strongly disagree  2 disagree  3 neutral  4 agree  5 strongly agree |
| **Subjective Norms** | **My parents and friends advised me to take the COVID-19 vaccine**  **Many people in my neighbourhood think that vaccination is an excellent way to prevent disease**  **I have seen people like me getting vaccinate** | 1 strongly disagree  2 disagree  3 neutral  4 agree  5 strongly agree |
| **Ability** | I am able to get the vaccine to avoid getting COVID-19  It is easy for me to get a vaccine to protect me from COVID-19 | 1 strongly disagree  2 disagree  3 neutral  4 agree  5 strongly agree |
| **Intention to receive COVID-19 vaccine** | I am seeking the COVID-19 vaccine  I will get vaccinated if my family or friends ask me to do so | 1 strongly disagree  2 disagree  3 neutral  4 agree  5 strongly agre |

**Table S2**. Socio-demographic characteristics of the HCWs and GP in Guinea.

| **Characteristic** | HCWs | GP |
| --- | --- | --- |
|  | **N = 3,547** | **N = 3,663** |
| Age |  |  |
| Young | 3,115 (88%) | 2,902 (79%) |
| Adult | 432 (12%) | 761 (21%) |
| Gender |  |  |
| Men | 1,339 (38%) | 2,542 (69%) |
| Women | 2,208 (62%) | 1,121 (31%) |
| Matrimonial status |  |  |
| Married | 1,935 (55%) | 1,711 (47%) |
| Single | 1,612 (45%) | 1,952 (53%) |
| Education |  |  |
| Second | 102 (2.9%) | 1,724 (47%) |
| University | 1,114 (31%) | 1,716 (47%) |
| High school | 2,331 (66%) | 223 (6.1%) |
| Occupation |  |  |
| Nurse assistant | 1,876 (53%) |  |
| Laboratory technician | 165 (4.7%) |  |
| Physician | 794 (22%) |  |
| Medical support | 95 (2.7%) |  |
| Midwife | 470 (13%) |  |
| Internship | 147 (4.1%) |  |
| Private-employee |  | 260 (7.1%) |
| Student |  | 896 (24%) |
| Civil-servant |  | 833 (23%) |
| Freelance |  | 1,434 (39%) |
| Unemployed |  | 240 (6.6%) |
| Household size |  |  |
| [1,5] | 1,562 (44%) | 1,753 (48%) |
| (5,10] | 1,428 (40%) | 1,362 (37%) |
| (10,30] | 557 (16%) | 548 (15%) |
| ≥ 18 years old | 3 (2, 5) | 3 (2, 5) |
| Length-stay |  |  |
| <6months | 362 (10%) | 404 (11%) |
| >=6months | 3,185 (90%) | 3,259 (89%) |
| Pregnancy |  |  |
| Yes | 152 (4.3%) | 77 (2.1%) |
| No | 2,054 (58%) | 1,040 (28%) |
| Not applicable | 1,341 (38%) | 2,546 (70%) |
| Household Income |  |  |
| High income | 266 (7.5%) | 442 (12%) |
| Low income | 512 (14%) | 475 (13%) |
| Middle income | 2,769 (78%) | 2,746 (75%) |

Legends:

- HCWs. Healthcare workers; GP. General population.
- Young: whose age is <40 and Adult: whose age is≥ 40.
- High income when the mean income is ≥ 2000000GNF and the number of people in the household is ≤10.
- Low-income households, when the mean income is < 2000000GNF and the number of people in the household is >10.
- All other cases were considered middle income.

**Table S3. Medical conditions reported by the HCWs and GP in Guinea.**

|  |  | |  | |
| --- | --- | --- | --- | --- |
| **Characteristic** | | HCWs | | GP |
|  | | **N = 3,547** | | **N = 3,663** |
| Diabetes |  | |  | |
| Yes | 83 (2.3%) | | 114 (3.1%) | |
| No | 3464 (97.7%) | | 3549 (96.9%) | |
| Hypertension |  | |  | |
| Yes | 154 (4.3%) | | 249 (6.8%) | |
| No | 3393 (95.7%) | | 3414 (93.2%) | |
| Obesity |  | |  | |
| Yes | 613 (17%) | | 897 (24%) | |
| No | 2934 (83%) | | 2766 (86%) | |
| Asthma |  | |  | |
| Yes | 123 (3.5%) | | 103 (2.8%) | |
| No | 3424 (96.5%) | | 3560 (97.2%) | |
| Other allergic conditions |  | |  | |
| Yes | 664 (19%) | | 649 (18%) | |
| No | 2883 (81%) | | 3014 (82%) | |
| Other chronic diseases |  | |  | |
| Yes | 336 (9.5%) | | 342 (9.3%) | |
| No | 3211 (80.5%) | | 3021(80.7%) | |

**HCWs. Healthcare workers ; GP. General population**

**Additional file 4. Factors related to COVID-19 and vaccine among HCWs and GP.**

|  | |  |  | |  |
| --- | --- | --- | --- | --- | --- |
| **Characteristic** | | |  | HCWs | GP |
|  | | |  | **N = 3,547** | **N = 3,663** |
| Vaccine knowledge | |  |  | |  |
| Yes | | 1,609 (45%) |  | | 1,769 (48%) |
| No | | 1938 (55%) |  | | 1894 (52%) |
| Seeking COVID vaccine news in the last three days | |  |  | |  |
| Yes | | 2,254 (64%) |  | | 2,025 (55%) |
| No | | 1293 (36%) |  | | 1638 (45%) |
| Perception | |  |  | |  |
| Positive | | 1,746 (49%) |  | | 1,774 (48%) |
| Negative | | 1,801 (51%) |  | | 1,889 (52%) |
| Negative attitude | |  |  | |  |
| Less negative | | 1,891 (53%) |  | | 1,961 (54%) |
| Much negative | | 1,656 (47%) |  | | 1,702 (46%) |
| Positive attitude | |  |  | |  |
| Less positive | | 2,603 (73%) |  | | 597 (16%) |
| Much positive | | 944 (27%) |  | | 3,066 (84%) |
| Norms | |  |  | |  |
| Less favourable | | 1,692 (48%) |  | | 1,927 (53%) |
| Favourable | | 1,855 (52%) |  | | 1,736 (47%) |
| Ability to get the vaccine | |  |  | |  |
| Not able | | 3,081 (87%) |  | | 1,340 (37%) |
| Able | | 466 (13%) |  | | 2,323 (63%) |
| Intend to get vaccinated | |  |  | |  |
| Less intend | | 2,401 (68%) |  | | 2,039 (56%) |
| More intend | | 1,146 (32%) |  | | 1,624 (44%) |
|  |  | | | | |

Legend. HCWs : vaccine knowledge scale (min=5,mean=7., max=10, scale≥mean=yes); perception (min=13, mean=43.6,max=65, scale≥mean=positive); negative attitude (min=7,mean=18.3, max=35, scale≥mean=much negative); positive attitude (min=2,mean=8.1,max=10, scale≥mean=much positive), norms (min=3,mean=10.6, max=15, scale≥mean=favourable), ability to get the vaccine (min=2,mean=7.6,max=10, scale≥mean=able), intend to get vaccinated (min=2,mean=6,max=10, scale≥mean=more intend). GP: ); perception (min=13, mean=42.7,max=65, scale≥mean=positive), negative attitude (min=7,mean=19.1, max=35, scale≥mean=much negative); positive attitude (min=2,mean=7.8,max=10, scale≥mean=much positive), norms (min=3,mean=10.2, max=15, scale≥mean=favourable); ability to get the vaccine (min=2,mean=6.8,max=10, scale≥mean=able), intend to get vaccinated (min=2,mean=6,4,max=10, scale≥mean=more intend).
