# Supplementary material for: Facilitators and barriers to COVID-19 vaccination among healthcare workers and the general population in Guinea
Source: BMC Infect Dis. 2022 Sep 27;22:752. doi: 10.1186/s12879-022-07742-3 (PMC9514191; doi:10.1186/s12879-022-07742-3)
Supplement: Supplementary file 6 — Additional file 6. Dataset supporting the conclusions of this article. [file 12879_2022_7742_MOESM6_ESM.pdf]

Facilitators and barriers to COVID-19 vaccination among healthcare workers and the general population in Guinea.

Almamy Amara TOURE[1,2], Fodé Amara TRAORE[3,4], Gnoume CAMARA[3], Aboubacar Sidiki MAGASSOUBA[5],Ibrahima BARRY[1], Mohamed Lamine KOUROUMA[4],Younoussa SYLLA[1], Naby Yaya CONTE[1,5], Diao CISSE[5], Nafissatou DIOUBATÉ[1], Sidikiba SIDIBE[6,7], Abdoul Habib BEAVOGUI[1], Alexandre DELAMOU[6,7].

Authors' affiliations

1Centre National de Formation et de Recherche en Santé Rurale de Mafèrinyah, Forécariah, Guinea. 2 Kofi Annan University of Guinea.Conakry,Guinea. 3 Faculté des Sciences et Techniques de la Santé, Université Gamal Abdel Nasser, Centre Hospitalo-Universitaire de Conakry,Service de maladies infectieuses. 4 Agence Nationale de Sécurité Sanitaire,Conakry,Guinea. 5 Centre MURAZ, Burkina Faso.6 Department of Public Health, Faculty of Sciences and Health Techniques, Gamal Abdel Nasser University, Conakry, Guinea. 7 African Centre of Excellence in the Prevention and Control of communicable Diseases (CEA-PCMT).

```
############################################################Loading COVID-19 data
cvhw1 <- read.csv2("D:\\MOOC\\cvhcw11.csv")
cvhw2 <- read.csv2("D:\\MOOC\\cvhcw22.csv",stringsAsFactors = FALSE)
cvgui <- read.csv2("D:\\MOOC\\guicv-19.csv",stringsAsFactors = FALSE)
vaccine <- read.csv2("D:\\MOOC\\vaccine-gui.csv",stringsAsFactors = FALSE)
############################################################
```

[1] Corresponding author
Almamy Amara TOURE, Centre National de Formation et de Recherche en Santé Rurale de Maferinyah, Forecariah, Guinea, almamy@maferinyah.org

```
library(dplyr)
library(survey)
library(party)
library(gtsummary)
library(car)
library(GGally)
library(forestmodel)
library(Epi)
library(ggeffects)
library(ggparty)
library(tidyverse)
library(ggparty)
library(na.tools)
library(lubridate)
library(ComplexUpset)
library(compareGroups)
library(lubridate)
library(questionr)

###########################################total healhcare workers=3388
###################################################################Recoding process
cvhw1 <- rbind(cvhw1,cvhw2)
rm(cvhw2)
cvhw1[c(3:7,10:11)] <- lapply(cvhw1[c(3:7,10:11)],factor)
##################Renaming variables names
dput(names(cvhw1))
names(cvhw1) <- c("today", "age", "gender",
        "matstatus", "residence",
        "education", "occupation",
        "hmembnum", "more18y",
```

"length-stay", "pregnant",

"diabetes", "hta", "obesity",

"asthma", "othallerg", "otherchrdis",

"knowd_vaccin", "vaccitypes",

"vaccin.mapi", "immunstatus",

"immuncollect", "seekcv19news",

"govradio", "private-radios",

"public-tv", "private-tv",

"social-media", "NAHS-website",

"neighborhood", "word-of-mouth",

"other-sources", "perception_peur_COVID.19.probable_attrap_COVID.19",

"perception_peur_COVID.19.arisque_COVID.19", "perception_peur_COVID.19.possible_attrap_COVID.19",

"perception_peur_COVID.19.pense_COVID.19_grav", "perception_peur_COVID.19.pense_COVID_19_cons_neg",

"perception_peur_COVID.19.pense_COVID.19_nuisibl", "perception_peur_COVID.19.trepeur_COVID_19",

"perception_peur_COVID.19.penser_COVID_19_malaise", "perception_peur_COVID.19.mains_moites_pense_COVID_19",

"perception_peur_COVID.19.peur_mourir_COVID.19", "perception_peur_COVID.19.regarde_nouvel_COVID.19_rend_nerveu",

"perception_peur_COVID.19.nepeut_dormir_COVID.19", "perception_peur_COVID.19.acc_rythmecard_palpitation_COVID.19",

"attitudes_croyance.prendr_vaccin_COVID.19_benefic", "attitudes_croyance.prendr_vaccin_COVID.19_renf_immun",

"attitudes_croyance.vaccins_reserv_grpes_pers", "attitudes_croyance.vaccins_tropcher",

"attitudes_croyance.procedur_avoirvacc_laborieu", "attitudes_croyance.peur_effet_second_vaccin",

"attitudes_croyance.appri_vaccin_donnemaladie", "attitudes_croyance.vaccin_empecher_reproduct",

"attitudes_croyance.pas_confiance_vaccin", "normes_subjectives.parents_amis_cons_prendr_vaccin",

"normes_subjectives.voisinage_pense_vacc_bon", "normes_subjectives.vu_gens_commoi_vacciner",

```
                "capacite.seras_mesure_avoir_vacc", "capacite.facile_avoir_vaccin",

                "Intention.quete_vaccin_COVID.19", "vaccine",

                "Intention.mevaccin_famill_entourage_accpt", "revenumensuel",

                "GPS.nom_commune", "GPS.commune_gps", "GPS._commune_gps_latitude",

                "GPS._commune_gps_longitude", "GPS._commune_gps_altitude",
"GPS._commune_gps_precision",

                "GPS.border")

###########################################################################Recoding

#####################Gender

levels(cvhw1$gender)

levels(cvhw1$gender) <- c("men","women")

table(cvhw1$gender)

#####################Residence

levels(cvhw1$residence)

levels(cvhw1$residence) <-
c("Matoto","Ratoma","Matam","Dixinn","Kaloum","Coyah","Kindia","Mamou","Kankan","Nzerokore","LabÃ©","DubrÃ©ka")

table(cvhw1$residence)

levels(cvhw1$residence) <-
c("Conakry","Conakry","Conakry","Conakry","Conakry","Kindia","Kindia","Mamou","Kankan","Nzerokore","LabÃ©","Kindia")

table(cvhw1$residence)


####################matrimonial status

levels(cvhw1$matstatus)

levels(cvhw1$matstatus) <- c("married","single","single","single")

table(cvhw1$matstatus)

################################Education

levels(cvhw1$education)

levels(cvhw1$education) <- c("second","second","second","univ","highscool")

table(cvhw1$education)
```

```
###################################occupation

levels(cvhw1$occupation)

levels(cvhw1$occupation) <-
c("hc2level","labtech","labtech","inf","inf","labtech","labtech","labtech","physician","adminwk","pharma
","midwife","midwife","internship")

table(cvhw1$occupation)

levels(cvhw1$occupation)

levels(cvhw1$occupation) <-
c("nurs.as","labtech","nurs.as","physician","medsupport","pharma","midwife","internship")

table(cvhw1$occupation)

levels(cvhw1$occupation)

levels(cvhw1$occupation) <-
c("nurs.as","labtech","physician","medsupport","medsupport","midwife","internship")

table(cvhw1$occupation)

#############################################################Recoding continued

levels(cvhw1$`length-stay`) <- c("<6months",">=6months")

table(cvhw1$`length-stay`)

levels(cvhw1$pregnant) <- c("yes","no")

table(cvhw1$pregnant)

cvhw1[c(12:17)] <- lapply(cvhw1[c(12:17)],factor)

levels(cvhw1$hta) <- levels(cvhw1$obesity) <- levels(cvhw1$diabetes) <- levels(cvhw1$othallerg) <-
levels(cvhw1$otherchrdis) <- levels(cvhw1$asthma)<- c("yes","no")

summary(cvhw1[c(12:17)])

#############################compute score values

attach(cvhw1)

cvhw1$knowd_vaccin<- knowd_vaccin+vaccitypes+vaccin.mapi+immunstatus+immuncollect

summary(cvhw1$knowd_vaccin)

cvhw1 <- cvhw1[-c(19:22)]

cvhw1$knowd_vaccin <- cut(cvhw1$knowd_vaccin,breaks = c(5,7.286,10),include.lowest = TRUE,labels
= c("no","yes"))

table(cvhw1$knowd_vaccin)
```

```
cvhw1$percep <- perception_peur_COVID.19.acc_rythmecard_palpitation_COVID.19+perception_peur_COVID.19.arisque_COVID.19+perception_peur_COVID.19.mains_moites_pense_COVID_19+perception_peur_COVID.19.nepeut_dormir_COVID.19+perception_peur_COVID.19.pense_COVID.19_grav+perception_peur_COVID.19.pense_COVID.19_nuisibl+perception_peur_COVID.19.pense_COVID_19_cons_neg+perception_peur_COVID.19.penser_COVID_19_malaise+perception_peur_COVID.19.peur_mourir_COVID.19+perception_peur_COVID.19.possible_attrap_COVID.19+perception_peur_COVID.19.probable_attrap_COVID.19+perception_peur_COVID.19.regarde_nouvel_COVID.19_rend_nerveu+perception_peur_COVID.19.trepeur_COVID_19

summary(cvhw1$percep)

cvhw1$percep <- cut(cvhw1$percep,breaks = c(13,43.6,65),include.lowest = TRUE,labels = c("pos","neg"))

table(cvhw1$percep)

cvhw1 <- cvhw1[-c(29:41)]


cvhw1$negatt <- attitudes_croyance.appri_vaccin_donnemaladie+attitudes_croyance.pas_confiance_vaccin+attitudes_croyance.peur_effet_second_vaccin+attitudes_croyance.procedur_avoirvacc_laborieu+attitudes_croyance.vaccin_empecher_reproduct+attitudes_croyance.vaccins_reserv_grpes_pers+attitudes_croyance.vaccins_tropcher

summary(cvhw1$negatt)

cvhw1$negatt <- cut(cvhw1$negatt,breaks = c(7,18.25,35),include.lowest = TRUE,labels = c("lessneg","muchneg"))

table(cvhw1$negatt)

cvhw1$posatt <- attitudes_croyance.prendr_vaccin_COVID.19_benefic+attitudes_croyance.prendr_vaccin_COVID.19_renf_immun

summary(cvhw1$posatt)

cvhw1$posatt <- cut(cvhw1$posatt,breaks = c(2,8.182,10),include.lowest = TRUE,labels = c("lesspos","muchpos"))

table(cvhw1$posatt)

cvhw1 <- cvhw1[-c(29:37)]

cvhw1$norm <- normes_subjectives.parents_amis_cons_prendr_vaccin+normes_subjectives.voisinage_pense_vacc_bon+normes_subjectives.vu_gens_commoi_vacciner

summary(cvhw1$norm)
```

```
cvhw1$norm <- cut(cvhw1$norm,breaks = c(3,10.61,15),include.lowest = TRUE,labels =
c("lessfav","favor"))

table(cvhw1$norm)

cvhw1 <- cvhw1[-c(29:31)]

cvhw1$abil <- capacite.facile_avoir_vaccin+capacite.seras_mesure_avoir_vacc

summary(cvhw1$abil)

cvhw1$abil <- cut(cvhw1$abil,breaks = c(2,8,10),include.lowest = TRUE,labels = c("notabl","able"))

table(cvhw1$abil)

cvhw1 <- cvhw1[-c(29:30)]

cvhw1$glint <- cvhw1$Intention.mevaccin_famill_entourage_accpt+Intention.quete_vaccin_COVID.19

summary(cvhw1$glint)

cvhw1$glint <- cut(cvhw1$glint,breaks = c(2,6,10),include.lowest = TRUE,labels =
c("lessint","goodint"))

table(cvhw1$glint)

cvhw1 <- cvhw1[-c(29,31)]


############################################################

cvhw1$gender <- na.resample(cvhw1$gender)

levels(cvhw1$vaccine)

cvhw1$vaccine <- factor(cvhw1$vaccine)

levels(cvhw1$vaccine) <- c("no","no","no","yes","yes")

table(cvhw1$vaccine)

cvhw1$seekcv19news <- factor(cvhw1$seekcv19news)

levels(cvhw1$seekcv19news) <- c("yes","no")

table(cvhw1$seekcv19news)


#########################################Imputation for income

cvhw1

set.seed(123)

cvhw1$income<-na.bootstrap(cvhw1$revenumensuel)

cvhw1$income<-na.bootstrap(cvhw1$income)
```

```
cvhw1$income<-na.bootstrap(cvhw1$income)
cvhw1$income<-na.bootstrap(cvhw1$income)
cvhw1$income<-na.median(cvhw1$income)
summary(cvhw1$income)
cvhw1 <- cvhw1[-c(30)]


#################################################For General population
############################################total healhcare workers=3388
#################################################################Recoding process
cvpub1 <- read.csv2("D:\\MOOC\\cvpub1.csv")
cvpub2 <- read.csv2("D:\\MOOC\\cvpub2.csv")
cvpub1 <- rbind(cvpub1,cvpub2)
rm(cvpub2)
cvpub1[c(3:7,10:11,18)] <- lapply(cvpub1[c(3:7,10:11,18)],factor)
###################Renaming variables names
dput(names(cvpub1))
names(cvpub1) <- c("today", "age", "gender",
            "matstatus", "residence",
            "education", "occupation",
            "hmembnum", "more18y",
            "length-stay", "pregnant",
            "diabetes", "hta", "obesity",
            "asthma", "othallerg", "otherchrdis",
            "knowd_vaccin", "seekcv19news",
            "government-radio", "private-radios",
            "public-tv", "private-tv",
            "social-media", "NAHS-website",
            "neighborhood", "word-of-mouth",
            "other-sources", "perception_peur_COVID.19.probable_attrap_COVID.19",
```

```
"perception_peur_COVID.19.arisque_COVID.19",
"perception_peur_COVID.19.possible_attrap_COVID.19",

"perception_peur_COVID.19.pense_COVID.19_grav",
"perception_peur_COVID.19.pense_COVID_19_cons_neg",

"perception_peur_COVID.19.pense_COVID.19_nuisibl",
"perception_peur_COVID.19.trepeur_COVID_19",

"perception_peur_COVID.19.penser_COVID_19_malaise",
"perception_peur_COVID.19.mains_moites_pense_COVID_19",

"perception_peur_COVID.19.peur_mourir_COVID.19",
"perception_peur_COVID.19.regarde_nouvel_COVID.19_rend_nerveu",

"perception_peur_COVID.19.nepeut_dormir_COVID.19",
"perception_peur_COVID.19.acc_rythmecard_palpitation_COVID.19",

"attitudes_croyance.prendr_vaccin_COVID.19_benefic",
"attitudes_croyance.prendr_vaccin_COVID.19_renf_immun",

"attitudes_croyance.vaccins_reserv_grpes_pers", "attitudes_croyance.vaccins_tropcher",

"attitudes_croyance.procedur_avoirvacc_laborieu",
"attitudes_croyance.peur_effet_second_vaccin",

"attitudes_croyance.appri_vaccin_donnemaladie",
"attitudes_croyance.vaccin_empecher_reproduct",

"attitudes_croyance.pas_confiance_vaccin",
"normes_subjectives.parents_amis_cons_prendr_vaccin",

"normes_subjectives.voisinage_pense_vacc_bon",
"normes_subjectives.vu_gens_commoi_vacciner",

"capacite.seras_mesure_avoir_vacc", "capacite.facile_avoir_vaccin",

"Intention.quete_vaccin_COVID.19", "vaccine",

"Intention.mevaccin_famill_entourage_accpt", "Intention.revenu_mensuel",

"GPS.nom_commune", "GPS.commune_gps", "GPS._commune_gps_latitude",

"GPS._commune_gps_longitude", "GPS._commune_gps_altitude",
"GPS._commune_gps_precision",

"GPS.border")

#####################################################################Recoding

#####################Gender

levels(cvpub1$gender)

levels(cvpub1$gender) <- c("men","women")
```

```r
table(cvpub1$gender)

#####################Residence

levels(cvpub1$residence)

levels(cvpub1$residence) <- c("Matoto","Ratoma","Matam","Dixinn","Kaloum","Coyah","Kindia","Mamou","Kankan","Nzerokore","LabÃ©","DubrÃ©ka")

table(cvpub1$residence)

levels(cvpub1$residence) <- c("Conakry","Conakry","Conakry","Conakry","Conakry","Kindia","Kindia","Mamou","Kankan","Nzerokore","Mamou","Kindia")

table(cvpub1$residence)


#####################matrimonial status

levels(cvpub1$matstatus)

levels(cvpub1$matstatus) <- c("married","single","single","single")

table(cvpub1$matstatus)

###############################Education

levels(cvpub1$education)

levels(cvpub1$education) <- c("second","second","second","univ","highscool")

table(cvpub1$education)


#############################occupation

levels(cvpub1$occupation)

levels(cvpub1$occupation) <- c("private-employee","student","student","civil-servant","civil-servant","freelance","unemployed","unemployed")

table(cvpub1$occupation)

levels(cvpub1$occupation)

##################################################Recoding continued

levels(cvpub1$`length-stay`) <- c("<6months",">=6months")

table(cvpub1$`length-stay`)

levels(cvpub1$pregnant) <- c("yes","no")

table(cvpub1$pregnant)
```

```
summary(cvpub1[c(12:23)])

levels(cvpub1$knowd_vaccin) <- c("yes","no")

table(cvpub1$knowd_vaccin)


cvpub1[c(12:17)] <- lapply(cvpub1[c(12:17)],factor)

levels(cvpub1$hta) <- levels(cvpub1$obesity) <- levels(cvpub1$diabetes) <- levels(cvpub1$othallerg) <- levels(cvpub1$otherchrdis) <- levels(cvpub1$asthma)<- c("yes","no")

summary(cvpub1[c(12:17)])


##############################Variables for recding

attach(cvpub1)

cvpub1$percep <- perception_peur_COVID.19.acc_rythmecard_palpitation_COVID.19+perception_peur_COVID.19.arisque_COVID.19+perception_peur_COVID.19.mains_moites_pense_COVID_19+perception_peur_COVID.19.nepeut_dormir_COVID.19+perception_peur_COVID.19.pense_COVID.19_grav+perception_peur_COVID.19.pense_COVID.19_nuisibl+perception_peur_COVID.19.pense_COVID_19_cons_neg+perception_peur_COVID.19.penser_COVID_19_malaise+perception_peur_COVID.19.peur_mourir_COVID.19+perception_peur_COVID.19.possible_attrap_COVID.19+perception_peur_COVID.19.probable_attrap_COVID.19+perception_peur_COVID.19.regarde_nouvel_COVID.19_rend_nerveu+perception_peur_COVID.19.trepeur_COVID_19

summary(cvpub1$percep)

cvpub1$percep <- cut(cvpub1$percep,breaks = c(13,42.7,65),include.lowest = TRUE,labels = c("pos","neg"))

table(cvpub1$percep)

cvpub1 <- cvpub1[-c(29:41)]


cvpub1$negatt <- attitudes_croyance.appri_vaccin_donnemaladie+attitudes_croyance.pas_confiance_vaccin+attitudes_croyance.peur_effet_second_vaccin+attitudes_croyance.procedur_avoirvacc_laborieu+attitudes_croyance.vaccin_empecher_reproduct+attitudes_croyance.vaccins_reserv_grpes_pers+attitudes_croyance.vaccins_tropcher

summary(cvpub1$negatt)

cvpub1$negatt <- cut(cvpub1$negatt,breaks = c(7,19.15,35),include.lowest = TRUE,labels = c("lessneg","muchneg"))

table(cvpub1$negatt)
```

```
cvpub1$posatt <-
attitudes_croyance.prendr_vaccin_COVID.19_benefic+attitudes_croyance.prendr_vaccin_COVID.19_ren
f_immun

summary(cvpub1$posatt)

cvpub1$posatt <- cut(cvpub1$posatt,breaks = c(2, 7.872,10),include.lowest = TRUE,labels =
c("lesspos","muchpos"))

table(cvpub1$posatt)

cvpub1 <- cvpub1[-c(29:37)]

cvpub1$norm <-
normes_subjectives.parents_amis_cons_prendr_vaccin+normes_subjectives.voisinage_pense_vacc_bon+n
ormes_subjectives.vu_gens_commoi_vacciner

summary(cvpub1$norm)

cvpub1$norm <- cut(cvpub1$norm,breaks = c(3,10.23,15),include.lowest = TRUE,labels =
c("lessfav","favor"))

table(cvpub1$norm)

cvpub1 <- cvpub1[-c(29:31)]

cvpub1$abil <- capacite.facile_avoir_vaccin+capacite.seras_mesure_avoir_vacc

summary(cvpub1$abil)

cvpub1$abil <- cut(cvpub1$abil,breaks = c(2,6.871,10),include.lowest = TRUE,labels =
c("notabl","able"))

table(cvpub1$abil)

cvpub1 <- cvpub1[-c(29:30)]

cvpub1$glint <- cvpub1$Intention.mevaccin_famill_entourage_accpt+Intention.quete_vaccin_COVID.19

summary(cvpub1$glint)

cvpub1$glint <- cut(cvpub1$glint,breaks = c(2,6.423,10),include.lowest = TRUE,labels =
c("lessint","goodint"))

table(cvpub1$glint)

cvpub1 <- cvpub1[-c(29,31)]

sapply(cvpub1, function(x)sum(is.na(x)))

cvpub1$gender <- na.resample(cvpub1$gender)

##############################################################

levels(cvpub1$vaccine)

cvpub1$vaccine <- factor(cvpub1$vaccine)
```

```
levels(cvpub1$vaccine) <- c("no","no","no","yes","yes")

table(cvpub1$vaccine)

cvpub1$seekcv19news <- factor(cvpub1$seekcv19news)

levels(cvpub1$seekcv19news) <- c("yes","no")

table(cvpub1$seekcv19news)

set.seed(123)

summary(cvpub1$Intention.revenu_mensuel)

cvpub1$income<-na.bootstrap(cvpub1$Intention.revenu_mensuel)

cvpub1$income<-na.bootstrap(cvpub1$income)

cvpub1$income<-na.bootstrap(cvpub1$income)

cvpub1$income<-na.bootstrap(cvpub1$income)

summary(cvpub1$income)

cvpub1 <- cvpub1[-c(30)]


####################################################################################################Merging datasets

#covac <- rbind(cvhw1,cvpub1)

#levels(covac$occupation)

#save.image("D:\\MOOC\\covax.RData")

#load("D:\\MOOC\\covax.RData")

summary(cvhw1$income)

summary(cvpub1$income)

table(cvhw1$hmembnum)

cvhw1$incomem[cvhw1$income>=2000000& cvhw1$hmembnum<=10] <- "high-inc"

cvhw1$incomem[cvhw1$income>=2000000& cvhw1$hmembnum>10] <- "middle-inc"

cvhw1$incomem[cvhw1$income<2000000& cvhw1$hmembnum<=10] <- "middle-inc"

cvhw1$incomem[cvhw1$income<2000000& cvhw1$hmembnum>10] <- "low-inc"

table(cvhw1$incomem)

cvhw1$incomem <- factor(cvhw1$incomem)

summary(cvhw1$age)
```

```
cvhw1$age2 <- cut(cvhw1$age,breaks = c(18,40,75),include.lowest = TRUE,labels = c("young","adult"))

table(cvhw1$age2)


cvpub1$incomem[cvpub1$income>=2000000& cvpub1$hmembnum<=10] <- "high-inc"

cvpub1$incomem[cvpub1$income>=2000000& cvpub1$hmembnum>10] <- "middle-inc"

cvpub1$incomem[cvpub1$income<2000000& cvpub1$hmembnum<=10] <- "middle-inc"

cvpub1$incomem[cvpub1$income<2000000& cvpub1$hmembnum>10] <- "low-inc"

table(cvpub1$incomem)

summary(cvpub1$age)

cvpub1$age2 <- cut(cvpub1$age,breaks = c(18,40,80),include.lowest = TRUE,labels =
c("young","adult"))

table(cvpub1$age2)

cvpub1$incomem <- factor(cvpub1$incomem)


################################################################

cvpub1$hmembnum <- cut(cvpub1$hmembnum,breaks = c(1,5,10,30),include.lowest = TRUE)

table(cvpub1$hmembnum)

summary(cvpub1$more18y)

summary(cvhw1$hmembnum)

cvhw1$hmembnum <- cut(cvhw1$hmembnum,breaks = c(1,5,10,30),include.lowest = TRUE)

table(cvhw1$hmembnum)

summary(cvhw1$more18y)

############################################################Replacing missing for pregnant
women

cvhw1$pregnant <- na.replace(cvhw1$pregnant,.na = "notapplic")

levels(cvhw1$pregnant)

cvpub1$pregnant <- na.replace(cvpub1$pregnant,.na = "notapplic")


#############################################recode variables for CART

levels(cvhw1$pregnant) <- c("yes","no","no")

levels(cvpub1$pregnant) <- c("yes","no","no")
```

```
levels(cvhw1$negatt) <- c("less-negative","more-negative")

levels(cvhw1$glint) <- c("less-intend","more-intend")

levels(cvhw1$norm) <- c("less-optimist","more-optimist")

cvhw1<- rename.variable(cvhw1,"negatt","negative-attitude")

cvhw1<- rename.variable(cvhw1,"pregnant","pregnancy")

cvhw1 <- rename.variable(cvhw1,"glint","intend.to.get.vaccinated")

cvhw1 <- rename.variable(cvhw1,"knowd_vaccin","vaccine.knowledge")

levels(cvpub1$abil) <- c("not.able","able")

levels(cvpub1$norm) <- c("less-optimist","more-optimist")

cvpub1<- rename.variable(cvpub1,"abil","ability.to.get.vaccine")

cvpub1<- rename.variable(cvpub1,"norm","norms")

levels(cvpub1$glint) <- c("less-intend","more-intend")

cvpub1 <- rename.variable(cvpub1,"glint","intend.to.get.vaccinated")


#####################################################################################Source of infos analysis

#####################################################################source of news

sourcenews=colnames(cvhw1)[20:28]

cvhw1[sourcenews] = cvhw1[sourcenews] == 1

t(head(cvhw1[sourcenews], 3))

#########################################################################################################count

upset(

  cvhw1, sourcenews, width_ratio=0.1,

  n_intersections=15

)


#################################################################################################adding percent

upset(

  cvhw1,
```

```
  sourcenews,
  base_annotations=list(
    'Intersection size'=intersection_size(
      text_colors=c(
        on_background='brown', on_bar='yellow'
      )
    )
    + annotate(
      geom='text', x=Inf, y=Inf,
      label=paste('Total:', nrow(cvhw1)),
      vjust=1, hjust=1
    )
    + ylab('Intersection size')
  ),
  min_size=10,
  width_ratio=0.2,
  n_intersections=15
)
########################################################################For Genaral Population
sourcenews=colnames(cvpub1)[20:28]


cvpub1[sourcenews] = cvpub1[sourcenews] == 1
t(head(cvpub1[sourcenews], 3))
##########################################################count
upset(
  cvpub1, sourcenews, width_ratio=0.2,
  n_intersections=15
)
```

```
################################adding percent
upset(
  cvpub1,
  sourcenews,
  base_annotations=list(
    'Intersection size'=intersection_size(
      text_colors=c(
        on_background='brown', on_bar='yellow'
      )
    )
    + annotate(
      geom='text', x=Inf, y=Inf,
      label=paste('Total:', nrow(cvpub1)),
      vjust=1, hjust=1
    )
    + ylab('Intersection size')
  ),
  min_size=10,
  width_ratio=0.2,
  n_intersections=15
)
#########################################################################Descriptive Analysis
cvhw11 <- cvhw1[-c(1,5,20:28,30:36,43)]
########################################Making graph for those vaccinated
library(lessR)
par(mfrow=c(1,2))
rm(vaccine)
tabbb1 <- BarChart(vaccine,data = cvhw11 ,fill="black", color="black", trans=.8, values_color="black", sort = "+")
```

```
###########################################################################
########################################################################
tabb1 <- tbl_summary(cvhw11)
table2 <-
  tbl_summary(
    cvhw11,
    by = vaccine, # split table by group
    missing = "no" # don't list missing data separately
  ) %>%
  add_n() %>% # add column with total number of non-missing observations
  add_p() %>% # test for a difference between groups
  modify_header(label = "**Variable**") %>% # update the column header
  bold_labels()
table2

###########
cvpub11 <- cvpub1[-c(1,5,20:28,30:36,43)]
######################################making graph
tabb2<- BarChart(vaccine,data = cvpub11 ,fill="blue", color="black", trans=.8, values_color="black",sort = "+")
tabb2 <- tbl_summary(cvpub11)
table3 <-
  tbl_summary(
    cvpub11,
    by = vaccine, # split table by group
    missing = "no" # don't list missing data separately
  ) %>%
  add_n() %>% # add column with total number of non-missing observations
  add_p() %>% # test for a difference between groups
  modify_header(label = "**Variable**") %>% # update the column header
```

```
  bold_labels()
table3
#########################################################################Merging descriptive table
tbl_merge(tbls = list(tabb1, tabb2)) %>%
  modify_spanning_header(everything() ~ NA_character_)
#############################################################################Univariate  data
tbl_merge(tbls = list(table2, table3)) %>%
  modify_spanning_header(everything() ~ NA_character_)


#################################################################################Multivariate analysis
cvhw11 <- cvhw11[-(1)]
cvhw1$vaccine <- relevel(cvhw1$vaccine,ref = "no")
levels(cvhw1$vaccine) <- c("0","1")
cvhw1$vaccine <- relevel(cvhw1$vaccine,ref = "0")
tab1 <- glm(vaccine~.,family = binomial(link = "logit"),data = cvhw11)
tab2 <- step(tab1)
######################################Model fit tools/Quality of the model healthcare workers
library(blorr)
blr_model_fit_stats(tab2)
##################confusion matrix
blr_confusion_matrix(tab2, cutoff = 0.5)
######################Goodness of fit Lemeshow
blr_test_hosmer_lemeshow(tab2)


##########################ROC Curve
tab2 %>%
  blr_gains_table() %>%
  blr_roc_curve()
```

```
############## Gains tables
tab2 %>%
  blr_gains_table() %>%
  blr_roc_curve()

###############KS chart should be big as (>=0.3)
tab2 %>%
  blr_gains_table() %>%
  blr_ks_chart()

###############Lorenz curve
blr_lorenz_curve(tab2)
################################################################################Model Evaluation
############## ROC curve
ROC(form=vaccine ~ gender + matstatus + education + occupation + `length-stay` +
      diabetes + knowd_vaccin + seekcv19news + percep + negatt +
      posatt + norm + glint + incomem + age2,plot="ROC",data = cvhw11)
#####################################################coefficients significance
anova(tab2, test = "Chisq")
##############model assessment
with(tab2,pchisq(null.deviance-deviance,df.null-df.residual,lower.tail = F))

##############################################mulitcollinearity
vif(tab2)##########no collinearity
###################################################################Building with gtsummary
tab2 <- tbl_regression(tab2,exponentiate = TRUE)
tab2
#############################################################Mulivariate analysis For population
cvpub11 <- cvpub11[-(1)]
```

```
cvpub1$vaccine <- relevel(cvpub1$vaccine,ref = "yes")
tab3 <- glm(vaccine~.,family = binomial(link = "logit"),data = cvpub11)
tab4 <- step(tab3)
#################Confusion matrix/ Quality of the model population
blr_model_fit_stats(tab4)
#################confusion matrix
blr_confusion_matrix(tab4, cutoff = 0.5)
###################Goodness of fit Lemeshow
blr_test_hosmer_lemeshow(tab4)

########################ROC Curve
tab4 %>%
  blr_gains_table() %>%
  blr_roc_curve()

############## Gains tables
tab4 %>%
  blr_gains_table() %>%
  blr_roc_curve()

##############KS chart should be big as (>=0.3)
tab4 %>%
  blr_gains_table() %>%
  blr_ks_chart()

##############Lorenz curve
blr_lorenz_curve(tab4)

############################################################Model Evaluation
############## ROC curve
```

```
ROC(form=vaccine ~ education + occupation + more18y +`length-stay`+
   hta + obesity + otherchrdis + knowd_vaccin + percep + `negative-attitude` +
   posatt + norm + abil + `intend.to.get.vaccinated` + age2,plot="ROC",data = cvpub11)
###################################################coefficients significance
anova(tab4, test = "Chisq")
##############model assessment
with(tab4,pchisq(null.deviance-deviance,df.null-df.residual,lower.tail = F))

#############################################mulitcollinearity
vif(tab4)##########no collinearity
tab4<- tbl_regression(tab4,exponentiate = TRUE)

#################################################################Merging Multivariate analysis
tbl_merge(tbls = list(tab2, tab4)) %>%
  modify_spanning_header(everything() ~ NA_character_)

#############################################################################Using tree decision
#A) For Healthcareworkers
tab10 <- ctree(vaccine ~ matstatus + occupation +education +pregnancy +
         vaccine.knowledge + seekcv19news + `negative-attitude` + norm + intend.to.get.vaccinated +
         incomem + age2,cvhw11)

nid <- nodeids(tab10)
iid <- nid[!(nid %in% nodeids(tab10, terminal = TRUE))]
(pval <- unlist(nodeapply(tab10, ids = iid,
               FUN = function(n) info_node(n)$p.value)))

myttnc2 <- nodeprune(tab10, ids = iid[pval > 1e-5])
```

```
plot(myttnc2)
tab10 <- as.constparty(myttnc2)

################################################Using###ggtree
ggparty(myttnc2) +
  geom_edge() +
  geom_edge_label() +
  geom_node_splitvar() +
  geom_node_plot(gglist = list(geom_bar(aes(x = "", fill = vaccine),
                                        position = position_fill()),
                               xlab("")),
                 # draw only one label for each axis
                 shared_axis_labels = TRUE,
                 # draw line between tree and legend
                 legend_separator = TRUE
  )
################################################################For population
tab11 <- ctree(vaccine ~ education + `length-stay` +
                 pregnant + hta + obesity + otherchrdis + knowd_vaccin + percep +
                 negatt + posatt + norms + ability.to.get.vaccine + intend.to.get.vaccinated + age2,cvpub11)

nid <- nodeids(tab11)
iid <- nid[!(nid %in% nodeids(tab11, terminal = TRUE))]
(pval <- unlist(nodeapply(tab11, ids = iid,
                          FUN = function(n) info_node(n)$p.value)))

myttnc3 <- nodeprune(tab11, ids = iid[pval > 1e-5])

plot(myttnc3)
tab11 <- as.constparty(myttnc3)
```

```
tab11 <- as.constparty(tab11)


######################################################################################Using
###ggtree

ggparty(tab11) +
  geom_edge() +
  geom_edge_label() +
  geom_node_splitvar() +
  geom_node_plot(gglist = list(geom_bar(aes(x = "", fill = vaccine),
                                        position = position_fill()),
                               xlab("")),
                 # draw only one label for each axis
                 shared_axis_labels = TRUE,
                 # draw line between tree and legend
                 legend_separator = TRUE
  )
```
